# Transfer RNA modifications and genes for modifying enzymes in *Arabidopsis thaliana*

**DOI:** 10.1186/1471-2229-10-201

**Published:** 2010-09-14

**Authors:** Peng Chen, Gunilla Jäger, Bo Zheng

**Affiliations:** 1 Qingdao Institute of Bioenergy and Bioprocess Technology, Chinese Academy of Sciences, China; 2 Department of Forest Genetics and Plant Physiology, Swedish Agricultural University, S-901 83, Umeå, Sweden; 3 Department of Molecular Biology, Umeå University, S-901 87, Umeå, Sweden

## Abstract

**Background:**

In all domains of life, transfer RNA (tRNA) molecules contain modified nucleosides. Modifications to tRNAs affect their coding capacity and influence codon-anticodon interactions. Nucleoside modification deficiencies have a diverse range of effects, from decreased virulence in bacteria, neural system disease in human, and gene expression and stress response changes in plants. The purpose of this study was to identify genes involved in tRNA modification in the model plant *Arabidopsis thaliana*, to understand the function of nucleoside modifications in plant growth and development.

**Results:**

In this study, we established a method for analyzing modified nucleosides in tRNAs from the model plant species, *Arabidopsis thaliana *and hybrid aspen (*Populus tremula *× *tremuloides*). 21 modified nucleosides in tRNAs were identified in both species. To identify the genes responsible for the plant tRNA modifications, we performed global analysis of the Arabidopsis genome for candidate genes. Based on the conserved domains of homologs in *Sacccharomyces cerevisiae *and *Escherichia coli*, more than 90 genes were predicted to encode tRNA modifying enzymes in the Arabidopsis genome. Transcript accumulation patterns for the genes in Arabidopsis and the phylogenetic distribution of the genes among different plant species were investigated. Transcripts for the majority of the Arabidopsis candidate genes were found to be most abundant in rosette leaves and shoot apices. Whereas most of the tRNA modifying gene families identified in the Arabidopsis genome was found to be present in other plant species, there was a big variation in the number of genes present for each family.

Through a loss of function mutagenesis study, we identified five tRNA modification genes (AtTRM10, AtTRM11, AtTRM82, AtKTI12 and AtELP1) responsible for four specific modified nucleosides (m^1^G, m^2^G, m^7^G and ncm^5^U), respectively (two genes: AtKTI12 and AtELP1 identified for ncm^5^U modification). The *AtTRM11 *mutant exhibited an early-flowering phenotype, and the *AtELP1 *mutant had narrow leaves, reduced root growth, an aberrant silique shape and defects in the generation of secondary shoots.

**Conclusions:**

Using a reverse genetics approach, we successfully isolated and identified five tRNA modification genes in *Arabidopsis thaliana*. We conclude that the method established in this study will facilitate the identification of tRNA modification genes in a wide variety of plant species.

## Background

**Figure 1:**
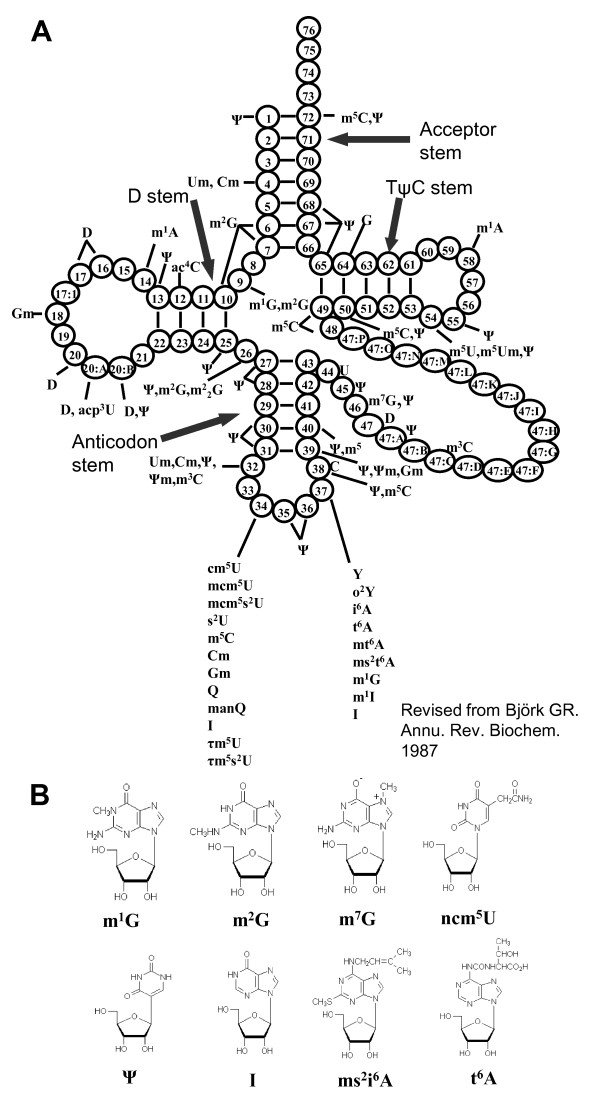
**Modified nucleosides in eukaryotic tRNAs and chemical structures**. A: Clover-leaf structure of eukaryotic tRNA. Each circle represents a nucleotide, numbered from 5'- to 3'- end. Modified nucleosides found at different positions are shown. B: Chemical structures of some modified nucleosides.

Transfer RNA (tRNA) is the adapter molecule mainly responsible for decoding mRNA into the corresponding peptide sequence. tRNA molecules are generally 75-87 nucleotides long and form clover-leaf shaped structures through base pairing in the acceptor stem; D-stem, TΨC stem and anticodon stem (Figure 1A). Modified tRNA nucleosides are found universally in living organisms. Some are conserved across all domains of life (e.g. Ψ, D, m^1^G, m^7^G, Cm, Um and Gm), indicating an evolutionary ancient enzyme [1]. According to the RNA modification database http://library.med.utah.edu/RNAmods/, 107 different modified nucleosides were found in RNA as at 2008. Among these, 92 are present on tRNA molecules. All modified nucleosides are derivatives of the four normal nucleosides: adenosine, guanosine, uracine and cytosine. The modifications vary from a simple methylation on the ribose or base moiety to complicated side chain modifications in different positions of the purine/pyramidine ring (Figure 1B).

All nucleoside modifications except Q are made on the polynucleotide level, i.e. they are made post-transcriptionally [2]. Many variations exist, however, in the regulation of modifications. For example, some eukaryotic tRNA modifications require intron-containing tRNA. Also, the modification of tRNAs can differ depending on the intracellular compartment, e.g. using yeast Phe-tRNA as a substrate in *Phaseolus vulgaris*, cytoplasmic and mitochondrial enzymes had m^5^C modification activity whereas chloroplast enzymes had m^1^A modification activity [3]. Modification pathways vary from a single methylation to complicated pathways involving multiple protein complexes, e.g. at least 25 gene products have been found to be involved in mcm^5^s^2^U modification in *S. cerevisiae *[4]. In *E. coli*, no modified nucleosides were shown to be essential for viability, however, the lack of certain modifying enzymes can lead to lethality [5]. In *S.cerevisiae*, three tRNA modifying enzymes (Gcd10p/Gcd14p, Tad2p/Tad3p and Thg1p) that modify m^1^A58 [6], I34 [7] and tRNA^His ^G_-1 _(guanine nucleotide to the 5'-end of tRNA^His^) [8] are known to be essential. Modified nucleosides influence the coding capacity of tRNA by strengthening or weakening anticodon-codon interactions and by influencing codon choice and codon context sensitivity. Deficiency of modified nucleosides can, therefore, lead to reduced translation efficiency and increased translation errors, which will affect gene expression regulation and cell metabolism [9]. Growth conditions and the environment can affect tRNA modifications both quantitatively and qualitatively, e.g. bacteria growing under starvation conditions for certain amino acids or iron leads to under-modification of tRNA [10]. The link between the synthesis of modified nucleosides in tRNA and metabolism has been suggested to be a regulatory device and tRNA modification as a "biological sensor". Studies in wheat have shown chromatographic changes of aminoacylated-tRNAs in different developmental stages [11] and an increase of Phe-tRNA Y(wybutosine) modification in older leaf tissues than in young leaf tissues [12]. Studies of different tissue types of tobacco showed that the abundance and variety of methylated nucleosides are greater in intact plants than in habituated and tumorous tissues [13]. tRNA modifications also differ upon maturation and/or transport into subcellular compartments such as mitochondria [14] or chloroplasts. Finally, some tRNA modifications require the presence of introns [15]. Clearly, therefore, which modified nucleosides are present on mature tRNA depends on when and where the modification occurs on the tRNA molecule.

Nucleoside modifications of tRNA have been extensively studied in bacteria and yeast and most of the biochemical pathways and genes encoding modification enzymes have been identified. By contrast, the study of tRNA modified nucleosides in plants has rarely been documented. As a result of their key role in the translation machinery, the mechanisms of regulation of tRNA activity by modified nucleosides are quite well-conserved. Some modified nucleosides are universally found in tRNAs from organisms of different domains of life, presumably because of their essential role for the structural stability of tRNA interactions with partner molecules during translation. In bacteria and *S. cerevisiae *tRNA, tRNA modifications have been suggested to act as biological sensors, changing quantity and quality according to the growth conditions. Plants encounter great environmental changes throughout their life cycles. This begs the question; do modified nucleosides change at different developmental stages, in different plant tissues or in response to environmental stimuli? How many modified nucleosides exist in plants, and how are they synthesized? These are the questions we want to investigate in order to understand the function of modified nucleotides in plant development. We chose *Arabidopsis thaliana *and hybrid aspen (*Populus tremula***× ***tremuloides*) for the study because both the Arabidopsis and the hybrid aspen genomes have been fully sequenced and because pools of mutants exist for Arabidopsis, facilitating the identification of genes for specific modified nucleosides. In addition, transgenic methods for both Arabidopsis and aspen are well-established. Hybrid aspen complements Arabidopsis because it is a perennial plant and therefore more suitable for study wood formation.

The methods used for RNA extraction and subsequent purification separate small RNAs (including tRNA, snRNA and miRNA) from high molecular weight RNA molecules (mRNA and rRNA). snRNA (small nuclear RNA) are extensively modified post-transcriptionally mainly by 2'-O-methylation and pseudouradylation at multiple positions. Modification in U2 snRNA from yeast and mammals have been shown to be important for the assembly and function of spliceosomes [16]. 2'-O-methylation of U2 snRNA has been shown to be conserved in plants but different from yeast and animals. The sequences of snoRNA which guide U2 snRNA modification by complementary sequences were also shown to be different between rice and Arabidopsis [17]. Plants have hundreds of miRNA genes and the abundance of miRNA might exceed tRNA under specific conditions (e.g. upon fungi infection). A considerable number of modifications (A to I editing and 2'-O-methylation of ribose,) are known to exist in plant miRNA [18]. The presence of modified nucleosides in plant tRNA is well accepted but modifying enzymes in plants has rarely been documented. One example of a modifying enzyme is the *ABO1/ELO2 *gene. Mutations in this gene, encoding a homolog of the yeast elongator complex protein, ELP1, can increase abscisic acid sensitivity and drought tolerance in Arabidopsis [19]. There are very few plant tRNA sequences available [20] for the identification of modified nucleosides on different positions of individual tRNA species, and very few plant tRNA modifying enzymes have been purified [21] or identified [22].

In this study we established a method for tRNA purification for the analysis of modified nucleosides in Arabidopsis and hybrid aspen (*Populus tremula ***× ***tremuloides*). Twenty one known and four novel modified nucleosides were detected in comparison with modified nucleosides found in other organisms. A combination-bioinformatics study and loss-of-function approach in Arabidopsis was used to identify five genes involved in modification of four specific modified nucleosides: m^1^G, m^2^G, m^7^G and ncm^5^U.

## Results

### 21 modified nucleosides and 4 novel nucleosides were detected in tRNAs of *Arabidopsis thaliana *and hybrid aspen

The model plants, *Arabidopsis thaliana *and hybrid aspen (*Populus tremula***× ***tremuloides*) were chosen for tRNA isolation and HPLC analysis. Because of the low yield of tRNA from plant tissues from previous experience, we used young seedlings of Arabidopsis and young leaves and shoot apices from hybrid aspen due to higher abundance of RNA in tissues of early developmental stages. From 5 g frozen tissue we were able to obtain approximately 1 mg total RNA using Trizol reagent. After removal of rRNA and mRNA by LiCl method we routinely obtained about 200 μg small RNA. From the last step of DE52 column purification about 40-50 μg tRNA were used for degradation and subsequent HPLC analysis. Gradient buffers consisting of three buffers were used to separate modified nucleosides and the elution time and spectrum of each peak were used to identify different modified nucleosides.

**Figure 2:**
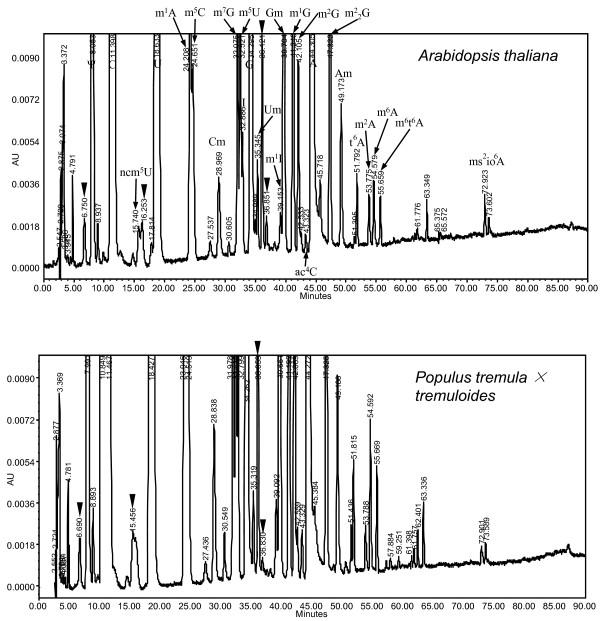
**HPLC chromatogram of modified nucleosides in tRNAs from Arabidopsis and Poplar**. X-scale: retention time of modified nucleosides in minutes. Y-scale: UV absorbance at 254 nm. Peaks marked with black triangle represent plant-specific modified nucleosides.

**Figure 3:**
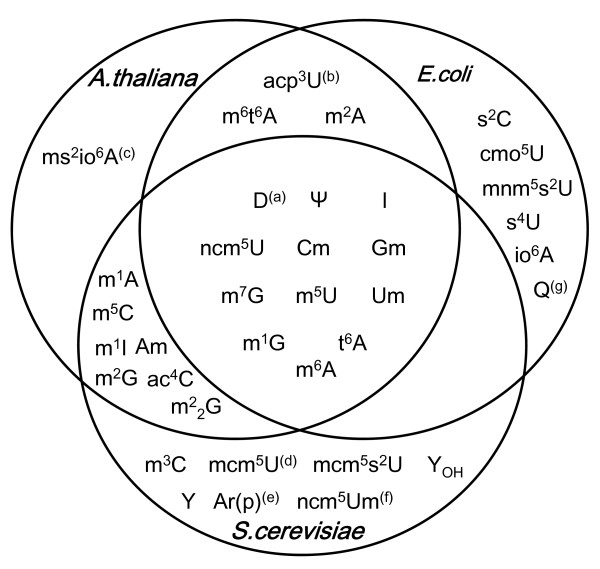
**Venn diagram showing similarities and differences of modified nucleosides between A. thaliana, S.cerevisiae and E. coli**. Modified nucleosides were shown with abbreviations, comments (a)-(g) were the same as in Table 1.

Twenty-one modified nucleosides were detected in Arabidopsis and hybrid aspen, listed according to the order of elution time from C30 column of HPLC analysis in Table 1. HPLC chromatograms of the two species were very similar (Figure 2); all modified nucleosides present in Arabidopsis were also present in hybrid aspen, with only slight differences for the relative abundance of some peaks. Dihydrouridine (D) is difficult to detect because it elutes together with Ψ, however D is well conserved and is the second most widely distributed modified nucleoside, therefore it should be present in plant tRNAs. Q was not analyzed because it is destroyed during the procedure used for tRNA extraction and digestion in this study. Q is present in *E. coli *and mammalian tRNA but absent in yeast tRNA[23]. Because the TGT gene responsible for Q biosynthesis is found in *P. trichocarpa *but not in *A. thaliana*, Q should be present in tRNA from hybrid aspen but absent in Arabidopsis. We compared the chromatogram with that from *S. cerevisiae*, calf liver and *E. coli *(Figure 3), certain prokaryotic tRNA modifications (e.g. s^2^C, s^4^U, mnm^5^s^2^U) were not found in plants, however m^2^A and ms^2^io^6^A which is present in bacteria but not in yeast and calf liver, was found in Arabidopsis and hybrid aspen tRNAs (Table 1). We also observed differences in tRNA modifications between plants, *S. cerevisiae *and calf liver. m^3^C and i^6^A are present in yeast tRNA but were not found in Arabidopsis and hybrid aspen. Genes for m^3^C modification were not identified. Several modified nucleosides (mcm^5^U, ncm^5^Um and Ar(p)) that were detected using purified single tRNA species from *S. cerevisiae *were not detected in this study. It is difficult to conclude whether these modified nucleosides were absent or of extremely low abundance. Wybutosine (Y) derivatives were not detected either, however, Arabidopsis genes involved in Y synthesis (At4g04670 and At1g75200) have been proposed [24].

**Table 1:** Modified nucleosides in Arabidopsis, Populus compared to *S. cerevisiae*.

Nucleosides^a^	Arabidopsis & Populus	*S. cerevisiae*	Calf liver	*E. coli*
D^b^	+^b^	+^b^	+^b^	+^b^
Ψ	+	+	+	+
C	+	+	+	+
cmo^5^U	-	-	-	+
ncm^5^U	+	+	+	+
U	+	+	+	+
m^3^C	-	+	+	-
s^2^C	-	-	-	+
m^1^A	+	+	+	-
m^5^C	+	+	+	-
mnm^5^s^2^U	-	-	-	+
Cm	+	+	+	+
m^7^G	+	+	+	+
m^5^U	+	+	+	+
I	+	+	+	+
G	+	+	+	+
Q^g^	n.a	-	+	+
acp^3^U	-	-	-	+
s^4^U	-	-	-	+
Um	+	+	+	+
m^1^I	+	+	+	-
mcm^5^U^c^	-	+	n.a.	n.a.
Gm	+	+	+	+
m^1^G	+	+	+	+
m^2^G	+	+	+	-
ac^4^C	+	+	-	-
A	+	+	+	+
m^2^_2_G	+	+	+	-
mcm^5^s^2^U	-	+	n.a.	n.a.
Am	+	+	+	-
t^6^A	+	+	+	+
m^2^A	+	-	-	+
m^6^A	+	+	+	+
m^6^t^6^A	+	-	+	+
Y_OH_	-	+	+	-
io^6^A	-	-	+	+
ms^2^io^6^A	+	-	-	-^f^
Y	-	+	-	-
Ar(p)^d^	n.a.	+	n.a.	n.a.
ncm^5^Um^e^	n.a.	+	n.a.	n.a.

a. Nucleosides are listed in the order of retention time in HPLC chromatogram, threshold for detection is approximately 0.002% of total area in HPLC chromatogram, any modified nucleoside below this threshold is designated as "-".

b. D (dihydrouridine) is not easily detected in HPLC system because of the very early retention time and because of its close elution with Ψ.

c. mcm^5^U is detected in yeast single tRNA prep but not in bulk tRNA preps.

d. Ar(p) is present on initiator tRNA in *S. cerevisiae *(Aström SU, 1994), its position in HPLC is unknown.

e. ncm^5^Um is detected in yeast single tRNA prep (Glasser AL, 1992), its position in HPLC is unknown.

f. *E. coli *has ms^2^i^6^A but *Salmonella enterica *has ms^2^io^6^A.

g. Q is not being analyzed because it is destroyed during tRNA extraction and digestion.

n.a. Data not available.

To summarize, four U derivatives, nine A derivatives, three C derivatives and five G derivatives were detected in a total of 21 modified nucleosides from Arabidopsis and hybrid aspen tRNAs. Four novel modified nucleosides were detected (marked with black triangles in Figure 2) and the identity of these plant-specific modified nucleosides requires further experimentation.

### Using bioinformatics to find tRNA modifying genes in plants

Many genes for tRNA modifying enzymes have been identified in yeast and bacteria (Table 2). We decided to look for tRNA modification genes by homology-based bioinformatics approaches [25]. We used protein sequences from *S. cerevisiae *or *E. coli *genes for the modified nucleosides detected in this study to find plant gene homologs from TAIR (The Arabidopsis Information Resource, http://www.arabidopsis.org) and NCBI databases http://www.ncbi.nlm.nih.gov/. Homologous genes are listed in Table 3 according to the order of modified nucleosides eluted from a C30 column from HPLC analysis. Phylogenetic trees for each family of genes were constructed using Geneious Basic 4.5.5 Tree Builder http://www.geneious.com based on protein sequences (Additional file 1).

**Table 2:** tRNA modification genes identified in *E. coli *and *S. cerevisiae*.

Modified nucleosides	*E. coli *genes	*S. cerevisiae *genes
D	DusA, DusB, DusC	Dus1, Dus2,Dus3,Dus4
Ψ	TruA/HisT(Ψ38,39,40), TruB(Ψ55), RluA(Ψ32), TruD(Ψ13)	Pus1(Ψ24,28,34,36), Pus3 (Ψ38,39), Pus4(Ψ55), Pus6(Ψ31), Pus7(Ψ13, Ψ35), Pus8(Ψ32), Pus9(Ψ32)
ncm^5^U		Sit4, Kti11-14, Elp1-6, Sap185, Sap190
m^1^A		Trm6, Trm61
m^5^C		Trm4
Cm	TrmJ(Cm32)	Trm7(Cm32)
m^7^G	YggH	Trm8, Trm82
m^5^U	TrmA	Trm2
I	TadA	Tad2, Tad3
Um	TrmJ(Um32)	Trm44(Um44)
m^1^I		Tad1, Trm5
mcm^5^U		Trm9, Sit4, Kti11-14, Elp1-6, Sap185, Sap190
Gm		Trm3(Gm18), Trm7(Gm34)
m^1^G	TrmD	Trm5, Trm10
m^2^G		Trm11, Trm112
ac^4^C	TmcA	TAN1
m^2^_2_G		Trm1
mcm^5^s^2^U	-	Urm1, Uba4,Ncs2, NFS1
Am		Trm13(Am4, Cm4)
t^6^A		Sua5
m^2^A	TrmG (genetic name, no sequence)	
m^6^t^6^A	TsaA	
i^6^A	MiaA	MOD5
ms^2^io^6^A	MiaA, MiaB, MiaE	
Y		Tyw1, Tyw2(Trm12), Tyw3, Tyw4
Ar(p)		Rit1
Q	Tgt, QueC,YbaX, YgcM, YgcF, YqcD	
preQ	QueF	
mnm^5^s^2^U	MnmE, MnmC, MnmA/AsuE/TrmU	
mnm^5^se^2^U	YbbB	
cmo^5^U	CmoA, CmoB	
mcmo^5^U	CmoA, CmoB	
k^2^C	MesJ/TilS	
s^2^U	TusA, TusB, TusC, TusD	
s^4^U	ThiI	
s^2^C	TtcA	Ncs6/Tuc1

**Table 3:** tRNA modification candidate genes in Arabidopsis.

Modified Nucleosides	Homologous genes found in Arabidopsis
D	At4g38890(Dus1,Dus2,Dus3), At5g67220(Dus1, Dus2, Dus3), At5g47970(Dus4), At3g49640(Dus1,Dus2,Dus3,Dus4), At3g63510(Dus1,Dus4)
Ψ	At1g76120cyto(Pus1,Pus3), At1g20370(Pus1,Pus3), At1g34150nucl(Pus3,Pus1), At3g06950chlo(Pus3,Pus1), At2g30320(Pus1,Pus3), At5g35400(Pus1,Pus3),At5g14460chlo(Pus4), At5g51140cyto(Pus6, Pus8, Pus9), At3g04820(Pus7), At3g52260cyto(Pus9,Pus8,Pus6), At1g76050chlo(Pus8, Pus9,Pus6), At3g19440(Pus9,Pus8,Pus6), At1g56345(Pus9), At1g78910(Pus9,Pus6)
ncm^5^U	At3g19980cyto, At1g50370cyto, At5g55260cyto, At4g26720cyto, At1g69960cyto, At3g58500cyto, At2g42500cyto, At1g10430cyto, At1g59830cyto (Sit4 family), At1g07990, At2g28360, At3g45190, At1g30470cyto(Sap185, Sap190), At2g15910(Kti11), At1g13870nucl(Kti12), At1g27060nucl, At5g63860nucl, At3g55580nucl, At5g16040, At3g53830nucl, At3g26100nucl (Kti13 family), At5g57015, At1g03930, At4g26100cyto, At1g72710 etc.(Kti14 family), At5g13680cyto(Elp1)
m^1^A	At5g14600(Trm61), At2g45730(Trm6)
m^5^C	At2g22400, At4g40000, At5g55920nucl, At4g26600chlo, At3g13180chlo, At1g06560, At5g66180, At5g26180(Trm4)
Cm	At5g01230, At4g25730, At5g13830cyto(Trm7)
m^7^G	At5g24840(Trm8), At5g17660chlo(Trm8), At1g03110nucl(Trm82)
m^5^U	At3g21300mito, At2g28450nucl(Trm2)
I	At1g48175, At1g68720nucl(Tad2), At5g24670nucl(Tad3)
Um	no gene homolog found
m^1^I	At1g01760nucl(Tad1),At3g56120cyto, At4g27340chlo, At4g04670cyto(Trm5)
Gm	At4g17610(Trm3), At5g01230, At4g25730, At5g13830cyto(Trm7)
m^1^G	At3g56120cyto, At4g27340chlo, At4g04670cyto(Trm5), At5g47680(Trm10)
m^2^G	At3g26410cyto(Trm11), At1g78190, At1g22270(Trm112)
ac^4^C	At5g12410(TAN1)
m^2^_2_G	At3g02320, At5g15810mito, At3g56330chlo(Trm1)
Am	At4g01880chlo(Trm13)
t^6^A	At5g60590chlo(Sua5)
ms^2^io^6^A	At4g36390chlo, At1g72090cyto(MiaB), At5g20040/ATIPT9, At2g27760/ATIPT2cyto(MOD5)
Y	At1g75200(Tyw1), At4g04670cyto(Tyw3+Tyw4c+Tyw2)

tRNA modifying genes from *S. cerevisiae *or *E. coli *were used for BLAST search in TAIR database to find homologous genes in *Arabidopsis thaliana*. Arabidopsis genes for each modified nucleosides detected in this study is listed and gene used in query was shown in bracket. For multiple gene homologs found in Arabidopsis the query gene is shown in bracket next to the last gene. Genes for m^2^A and m^6^A modification were not available, TsaA for m^6^t^6^A did not give any homologous genes in Arabidopsis. Predicted subcellular localization of gene products was shown when at least two prediction programs gave the same result (Additional file 2).

Dihydrouridine (D) and pseudouridine (Ψ) modification genes belong to dihydrouridine synthase superfamily or pseudouridine synthase superfamily, respectively. Dus1p-Dus4p are required for D modification at six different positions in yeast tRNA [26]. Dus3p homologs in plants were well grouped, less well for Dus1p homologs and no grouping was obvious for Dus2p and Dus4p (Additional file1). Pseudouridine is the most widely distributed modified nucleoside. It has been identified at 15 different positions on yeast tRNA [27]. In total, almost 100 homologous genes were found in plants, which code for modification enzymes responsible for Ψ at different locations on plant tRNA (Additional file1). To completely understand the differences between these gene homologs requires more phylogenetic and motif analyses and will not be investigated in this study.

**Figure 4:**
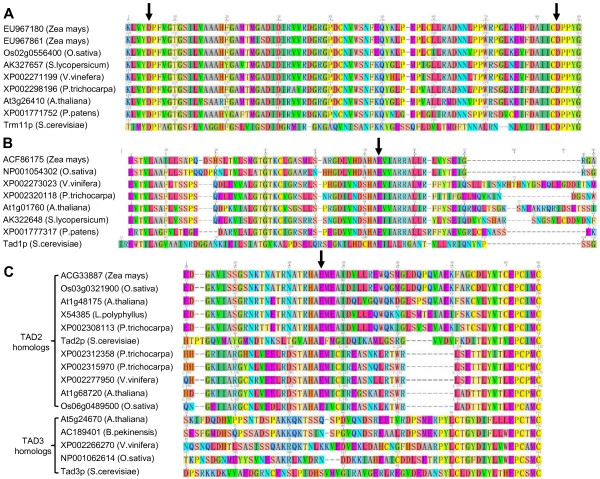
**Conserved domain of TRM11TAD1TAD2and TAD3 gene homologs in plants**. Part of protein sequence alignments were shown with numbers above showing position from the first amino acid. A: Motif I within catalytic domain of *TRM11 *gene homologs, conserved residue D215 and D291 are marked with arrows. B: Deaminase domain of *TAD1 *gene homologs, conserved residue is marked with black arrow. C: Deaminase domain of *TAD2 *and *TAD3 *gene homologs, conserved residue is marked with black arrow.

Methylation is the most common RNA modification, many methylated modified nucleosides exist in plant tRNA (m^1^G, m^2^G, m^2^_2_G, m^7^G, m^5^U, m^5^C, m^1^A, m^1^I, Am, Cm, Um and Gm, etc.). m^1^G is one of the most conserved modifications in tRNA. Trm5p and Trm10p are enzymes involved in the modification of m^1^G at different positions in *S. cerevisiae*. Although carrying similar biochemical activity, these two proteins do not share homology and are likely unrelated [28]. The Trm5p enzyme for m^1^G37 modification is an ancient protein. It is also involved in m^1^I modification [29]. Three *TRM5 *gene homologs and one *TRM10 *gene homolog were found in Arabidopsis and gene homologs are widely distributed in other plant species. Trm11p and Trm112p are both required for m^2^G modification in yeast tRNA [30]. One *TRM11 *and two *TRM112 *gene homologs were found in Arabidopsis. Conserved residues D215 of motif I and D291 of motif IV which are crucial for Trm11p catalytic activity [30] were conserved in all plant *TRM11 *gene homologs (Figure 4A). *TRM1 *codes for tRNA(m^2^_2_G) methyltransferase in *S. cerevisiae *[31]. Three Arabidopsis *TRM1 *gene homologs were found. Plant *TRM1 *gene homologs were divided into two groups (Additional file1). Trm8p and Trm82p form protein complexes required for m^7^G modification [32]. Two *TRM8 *gene homologs were found in Arabidopsis and plant *TRM8 *gene homologs can be divided into two groups. Plant *TRM82 *gene homologs are recognized as WD40-domain proteins (the same domain was found in Trm82p) which confer a wide variety of functions. m^5^U is one of the most conserved modified nucleosides, Trm2p protein contains tRNA(m^5^U) methyltransferase activity in *S. cerevisiae *[33]. In Arabidopsis, two *TRM2 *gene homologs were found. The yeast Trm4p protein catalyzes formation of m^5^C at positions 34, 40, 48 and 49 [34]. Eight *TRM4 *gene homologs were found in Arabidopsis belonging to the NOP1/NOP2/Sun protein family. Trm6 and Trm61 are essential genes coding for the two subunits of tRNA(m^1^A58) methyltransferase in yeast. One homolog was found in *Arabidopsis thaliana *for *Trm6 *and *Trm61*, respectively. m^1^I modification requires two gene products in yeast, Trm5p for methylation and Tad1p for deamination of A [35]. *TRM5 *homologs have been mentioned above. The Tad1p protein contains a deaminase domain and the conserved residue, E103, is maintained in all plant *TAD1 *gene homologs (Figure 4B).

In addition to base methylation, ribose methylation requires another group of methyl-transferases. Trm13p is responsible for Am and Cm modification at position 4 in *S. cerevisiae *[36]. Trm13p does not share obvious homology with other methyltransferases, plant *TRM13 *gene homologs all contain the TRM13 superfamily domain. One Arabidopsis *TRM13 *gene homolog was found, however, we failed to detect decreased amounts of Am in T-DNA knock-out mutants of this gene (data not shown). The Trm7p protein is responsible for both Cm32 and Gm34 modification in yeast [37]. Three *TRM7 *gene homologs were found in Arabidopsis. Trm44p was identified recently as tRNA(Um44) methyltransferase in *S. cerevisiae *[38]. Although Um was detected in Arabidopsis and Poplar tRNAs in this study, *TRM44 *gene homolog were not found. *TRM3 *gene is responsible for Gm18 modification [39]. One *TRM3 *gene homolog was found in Arabidopsis; however, once again we did not find any change of Gm content in a T-DNA knockout mutant carrying an insertion in an exon of this gene. This may be due to the presence of Gm at other positions.

At least 13 proteins have been shown to be involved in ncm^5^U modification in *S. cerevisiae *[4]. Elp1-6 are components of the elongator complex which are also involved in ncm^5^U modification by unknown mechanisms. Sit4p, Sap185p, Sap190p and Kti12p are a group of proteins that affect the phosphorylation status of Elp1 protein [40]. One Elp1 homolog was found in Arabidopsis and a few were identified in other plant species. Interestingly the Arabidopsis *abo1*(Elp1) mutant has been shown to be more resistant to drought and oxidative stress [19]. Sit4p belongs to the calcuneurin-like phosphoesterase protein family and 26 *SIT4 *gene homologs were found in Arabidopsis. Four Arabidopsis genes were found to be Sap185p and Sap190p homologs. Kti11-14 proteins are involved in resistance to *K. lactis *killer toxin of *S. cerevisiae *[41]: Kti13p belongs to the RCC1 family (regulator of chromosome condensation family) involved in regulating chromatin partitioning and cell division; Kti14p belongs to the Casein Kinase I-like protein family and physically interacts with the Elongator complex [4]. One Arabidopsis gene was found for Kti11p, one for Kti12p, six were found for Kti13p and around 90 homologs were found for Kti14p.

Inosine is a common modified nucleoside found in tRNAs. In *S. cerevisiae *Tad2p and Tad3p are subunits of adenosine deaminase for I34 formation [7]. Both proteins contain a deaminase domain and position E56 in Tad2p which is important for activity was retained in all plant *TAD2 *homologs (Figure 4C). Tan1p is responsible for ac^4^C modification in yeast [42] and one Arabidopsis *TAN1 *homolog was found. Plant *TAN1 *homologs can be divided into two groups (Additional file1). The *SUA5 *gene has been identified as a tRNA(t^6^A) synthase [25]. One *SUA5 *homolog was found in Arabidopsis and several were identified in other plants. ms^2^io^6^A modifications have two side chains: the ms^2^-group requires the MiaB protein in *S. enterica *and *E. coli *[43] and for i^6^- group modification, the *MOD5 *gene is required in *S. cerevisiae *[44]. The MiaE protein is required for modifying i^6^A to io^6^A in *S. enterica*. We found ms^2^io^6^A present in both Arabidopsis and hybrid aspen tRNAs. Two *MiaB *gene homologs were found in Arabidopsis and nine isopentenyl-transferases (ATIPT) have been identified in Arabidopsis, however, only two (ATIPT2 and ATIPT9) use tRNA as substrate [22]. No *MiaE *homologs were found in Arabidopsis.

**Figure 5:**
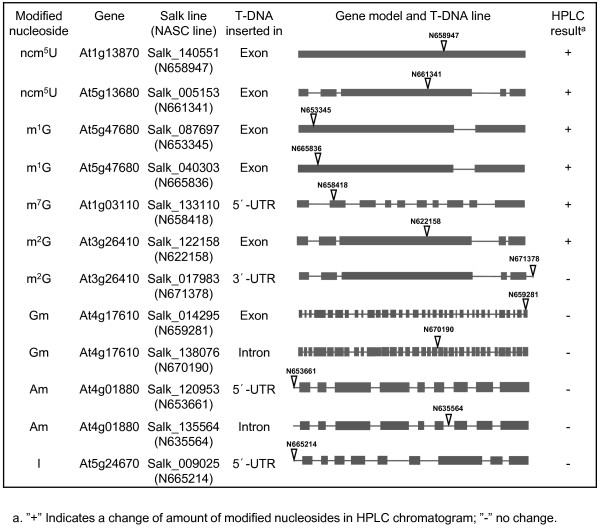
**T-DNA lines used in this study and corresponding genes**. Gene models were shown with dark gray box representing exon and lines in between as intron. T-DNA insertion was shown as a triangle with NASC line name above. Relevant modified nucleosides for corresponding gene and HPLC results were indicated: "+" represents a change of amount of modified nucleoside in the mutant; "-" represents no change.

**Figure 6:**
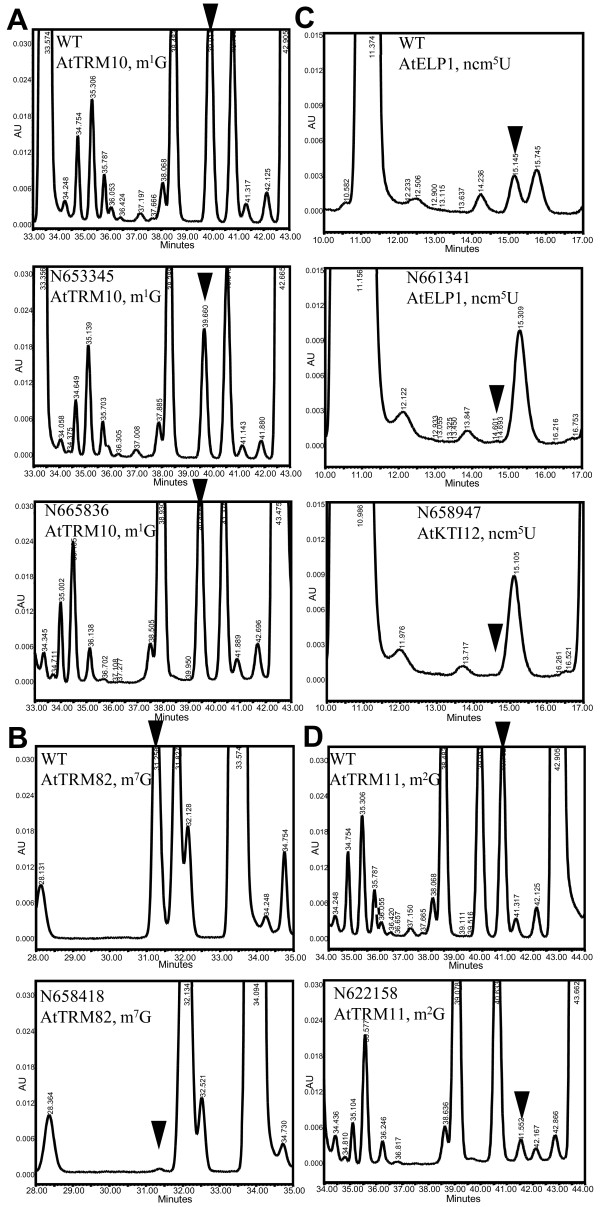
**HPLC chromatogram of the T-DNA homozygous mutants defective in modified nucleosides**. Parts of the HPLC chromatogram were shown with black triangle indicating position of the relevant modified nucleosides. NASC line number, allele number and modified nucleosides affected were shown in each panel, numbers above or within peaks represent retention time in minutes.

Based on the tRNA modification candidate genes found in Arabidopsis (Table 3), we decide to use publically available T-DNA mutant lines to identify genes specific for each modified nucleoside in Arabidopsis. We have chosen the genes of small gene families for which less than three genes were potentially involved in a certain modifications. 21 T-DNA insertional mutant lines were ordered from the European Arabidopsis Stock Center (NASC, http://arabidopsis.info/) for 13 genes involved in nine different modified nucleosides. Homozygote lines were isolated and modified nucleosides in total tRNA were subsequently analyzed. Twelve homozygous T-DNA lines were isolated, among them six lines were defective in four specific modified nucleosides: m^1^G, m^2^G, m^7^G and ncm^5^U (Table 4), T-DNA lines and their insertion sites are shown schematically in Figure 5. T-DNA lines in gene At5g47680 (Trm10p homolog) showed a 50% decrease in m^1^G content compared to wild type plants (Figure 6A). We named this gene AtTRM10. No m^7^G could be detected in plants with a T-DNA insertion in gene At1g03110 (Figure 6B). This gene is homologous to Trm82p. At1g03110 was named AtTRM82. Mutant plants from T-DNA NASC lines N661341 and N658947 showed no detectable ncm^5^U (Figure 6C). The corresponding genes, At5g13680 and At1g13870 were named AtELP1 and AtKTI12. At3g26410 was homologous to Trm11pthe gene required for m^2^G modification in *S. cerevisiae*. Only 7.3% of m^2^G remained in mutant plants compared to wild type plants (Figure 6D), At3g26410 was named AtTRM11.

**Table 4:** Quantification of modified nucleosides in T-DNA mutants.

T-DNA line (NASC line)	Gene	m^1^G/Ψ	m^7^G/Ψ	ncm^5^U/Ψ	m^2^G/Ψ
wt		0.344	0.338	0.014	0.273
N653345	At5g47680 (AtTRM10)	0.185 (53%)			
N665836	At5g47680 (AtTRM10)	0.208 (60%)			
N658418	At1g03110 (AtTRM82)		< 0.001 (< 0.2%)		
N661341	At5g13680 (AtELP1)			< 0.0002 (< 0.01%)	
N658947	At1g13870 (AtKTI12)			< 0.0003 (< 0.02%)	
N622158	At3g26410 (AtTRM11)				0.020 (7.3%)

Amount of certain modified nucleosides were shown using relative quantification to internal standard (Ψ), percentage of wild type level was shown in bracket.

Subcellular localization of tRNA modifying enzymes is an important issue, tRNA molecules are distributed in different subcellular compartments therefore modified nucleosides differ in mitochondria, chloroplast and cytoplasm. We performed prediction of protein subcellular localization using three programs: TargetP, WoLFPSORT and ESLpred (Additional File 2). The results from the three prediction program complement each other because different algorisms were used. Distribution of plant tRNA modification in different subcellular organelles is one of the future work to do, however we need to be cautious about cross-contamination to avoid false-positives because some of the modified nucleosides are present in low abundance.

### tRNA modifications are involved in regulating organ growth, stress responses and flowering time in *Arabidopsis thaliana*

Among the five genes identified in this study, two genes showed phenotype in the knock-out mutants. AtTRM10 and AtTRM82 mutants which showed dramatic decrease of m^1^G (Figure 6A) and m^7^G (Figure 6B) modified nucleosides respectively, did not show any phenotype under LD conditions. The AtKTI12 mutant, which carries a T-DNA inserted in an exon of At1g13870 similar to the previously isolated *drl1 *mutant [45] showed no detectable ncm^5^U (Figure 6C), however, narrow leaves and meristem defect phenotypes in *drl1 *mutant were not observed in the AtKTI12 mutant.

**Figure 7:**
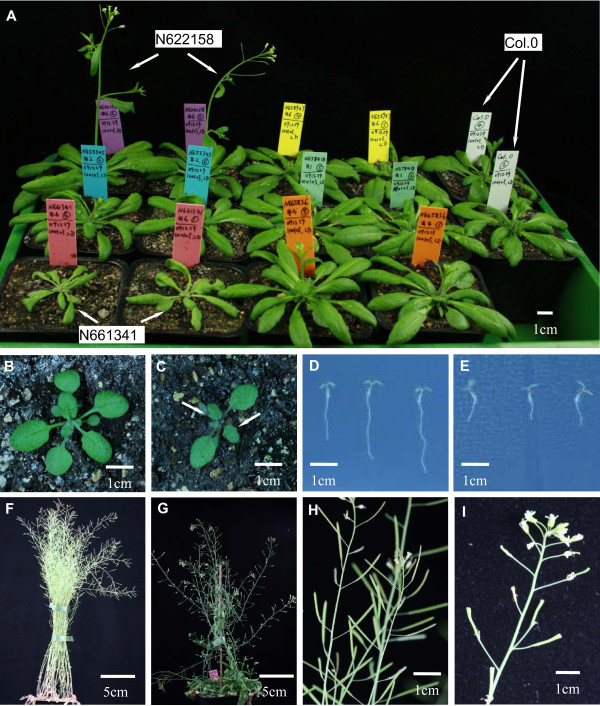
**Phenotype of mutant plants**. A: Early flowering of N622158 mutant plants and narrow leaf phenotype of N661341 mutant plants. Picture was taken at 21 D under LD condition. B and C: Serrated leaf shape of the third and fourth true leaves of N661341 mutant plant (C, indicated with arrows) compared to Col.0 (B). Picture was taken 13 D under LD condition. D and E: Reduced root growth of N661341 mutant plants (E) compared with Col.0 (D). MS plates were incubated vertically in tissue culture room for 8 D before picture was taken. F and G: N661341 mutant plant had problem of secondary shoot growth (G) compared to wild type (F). Plants were grown in LD conditions, primary shoots were cut at 3 weeks and picture taken at 6 weeks. H and I: N661341 mutant plant had aberrant silique shape (I) compared with Col.0 (H). Plants were grown in LD conditions.

The AtELP1 mutant, which carries a T-DNA insertion in the third exon of At5g13680, similar to the previously identified *elo2 *mutant[46] showed no detectable ncm^5^U (Figure 6C). The *elo2 *mutant belongs to the *elongata *mutants that have pleitrophic phenotypes, generally identified as reduced organ growth: narrow leaf, reduced growth of primary roots, altered inflorescence architecture and reduced length, delayed seeding growth [46]. The *elo2/abo1 *mutant also showed increased resistance to drought and oxidative stress, hypersensitivity towards ABA and elevated expression of anthocyanin biosynthesis genes[19,47]. The AtELP1 protein can complement the yeast *Δelp1 *mutant [19] and physically interacts with AtKIT12 [46]. The AtELP1 mutant in this study showed a narrow leaf shape (Figure 7A), and also reduced leaf numbers compare to wild type plants (Figure 7B) and serrated leaf edges of the third and fourth true leaves (Figure 7C). These phenotypes were also observed under short-day conditions (data not shown). AtELP1 mutant plants showed reduced root growth on MS medium plate (Figure 7E) compared to wild type plants (Figure 7D). AtELP1 mutants had reduced lateral shoot growth after the removal of the primary shoot. Lateral shoots had difficulties with remaining erect due to a defect in vascular tissues (Figure 7G). Finally, silique morphology was aberrant in this mutant (Figure 7I).

The AtTRM11 mutant, which carries T-DNA insertion in the third exon of At3g26410, showed a small amount (7.3% of wild type level) of m^2^G (Figure 6D). Under LD conditions the AtTRM11 mutant plant showed an early-flowering phenotype (Figure 7A) as well as reduced root growth on MS medium plates (Figure 7C). In *S. cerevisiae*, Trm112p is needed for m^2^G modification by regulating Trm11p activity [30]. No T-DNA lines are available for the two *TRM112 *homologs in Arabidopsis. Modifying enzymes for m^2^G at other positions have not been reported.

### Analysis of gene expression and phylogeny

**Figure 8:**
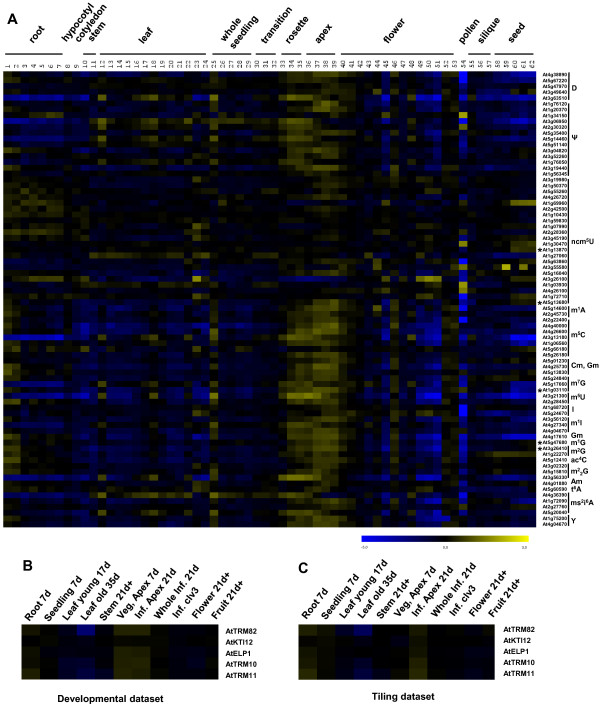
**Heat map of Arabidopsis tRNA modification candidate genes**. A: All tRNA modification candidate genes in Arabidopsis found in this study. Data downloaded from AtGenExpress database where information for tissue cluster and sample ID can be found. The five tRNA modification genes identified in this study were marked with asterisk. B and C: Heat map of AtTRM82, AtKTI12, AtELP1, AtTRM10 and AtTRM11 from Developmental dataset (B) and Tiling dataset (C) from Tileviz database.

We have investigated the expression pattern of all the Arabidopsis tRNA modification candidate genes identified in this study using the AtGenExpress database (Figure 8). 62 tissue samples were included. The candidate genes were grouped according to predicted function in specific modified nucleosides and mean-normalized expression values from the AtGenExpress database were transformed into log values for heat map construction using MeV (MultiExperiment Viewer) software. Most genes had prominent expression in rosette leaves and apex tissues, except for the D and ncm^5^U modification genes. The *AtTRM10, AtTRM11, AtTRM82, AtKTI12 *and *AtELP1 *genes identified in this study are marked with an asterisk. From the Tilviz database, except for AtKTI12, their expression was highest in apex tissues, and the expression level was higher in inflorescence apices than in vegetative apices according to the tiling dataset (Figure 8). AtKTI12 is only expressed in late stages of seed development. The expression heat map is a guideline for developmental and tissue specific expression of the candidate genes. The expression profile is very important for functional study. If the phenotype of the transgenic plants is consistent with the expression pattern, the following-up experiments will be performed in the right tissues and at the right developmental stages.

**Figure 9:**
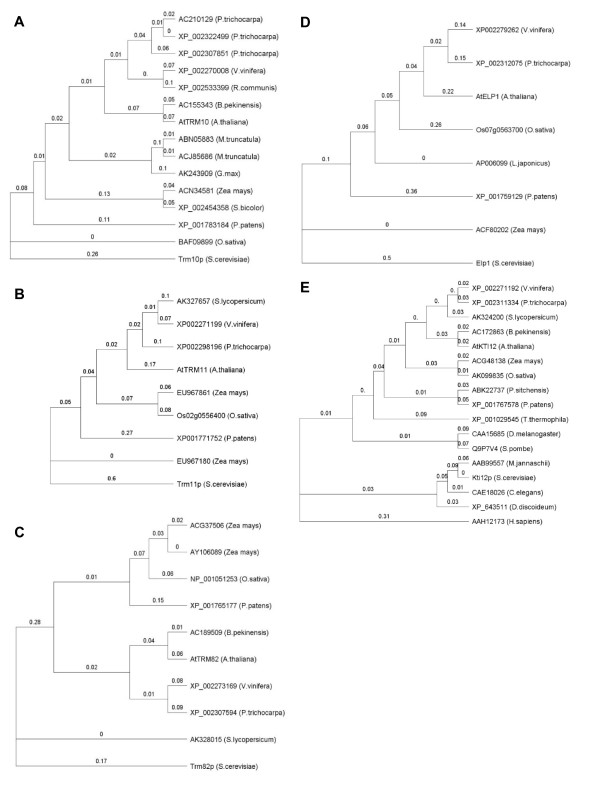
**Phylogenetic tree of TRM10, TRM11, TRM82, ELP1 gene homologs in plants and KTI12 tree from all domains of life**. Gene accession number and organism was shown, with branch numbers showing substitution rate per site for sequence alignment. A: Trm10 tree; B: Trm11 tree; C: Trm82 tree; D: Elp1 tree. E: Kti12 tree (representative of organism from all domains of life).

We searched for homologs for the 21 modified nucleosides and dihydrouridine present in Arabidopsis and hybrid aspen. Arabidopsis tRNA modification candidate genes are listed in Table 3. An unrooted Neighbour-Joining tree was constructed showing the phylogeny between plant genes in relation to yeast genes (Figure 9). The result shows that most gene families in Arabidopsis also exist in other plants; however, the number of genes in each family varies. In this section we will discuss only the homologs for the five Arabidopsis tRNA modification genes identified in this study: i.e. the homologs for AtTRM10, AtTRM11, AtTRM82, AtKTI12 and AtELP1. Trm10p is a conserved methyl-transferase, but it does not contain a typical AdoMet-binding domain and shares no homology with other classical tRNA methyltransferases, e.g. Trm5p [28]. Fourteen plant *TRM10*homologs were found, including one Arabidopsis gene (AtTRM10), two from *M. truncatula *and three from *P. trichocarpa *(Figure 9A). Trm11p is required for m^2^G modification in yeast tRNA. The residues D215 and D291 that are essential for Trm11p catalytic activity are retained in all plant *TRM11 *gene homologs, including AtTRM11 (Figure 4A). Trm82p belongs to the WD40-domain protein family. Members of this protein family have different biological functions. AtTRM82 is clearly responsible for m^7^G modification in *Arabidopsis thaliana*. Elp1 and Kti12p are well conserved proteins and both are involved in ncm5U modification. Only a few ELP1 homologs were found in plants (Figure 9D), in contrast to the *KTI12 *homologs that are found from archea to human (Figure 9E).

The majority of tRNA modification enzymes are not essential, in bacteria only TrmA enzyme has been shown to be essential, however the lethality is not due to lack of m^5^U modification on tRNA but from its effect on ribosome assembly by association with rRNA. In yeast three tRNA modifying enzymes/complexes were found to be essential: Gcd10p/Gcd14p, Tad2p/Tad3p and Thg1p. At present we could not conclude which plant genes encoding tRNA modification enzymes are essential, however during the preliminary screening of the T-DNA lines for tRNA modification genes in Arabidopsis we were not able to isolate homozygote plant from some of the lines (n≥24, n represent number of plants used), which is probably due to essentiality of the corresponding genes. To confirm this, we will increase the amount of plants for screening in the next generation (n≥200), at the same time we will carry out more comprehensive identification and confirmation of the tRNA modification candidate genes.

## Discussion

In this study we investigated modified nucleosides in the model plant species *Arabidopsis thaliana *and hybrid aspen. Twenty one modified nucleosides were detected in young tissues from both species. The method used did not allow for dihydrouridine (D) and queosine (Q) derivatives to be analyzed. However as D is much conserved in tRNAs from all domains of life, we assume that D is also present in plant tRNAs. The TGT gene responsible for Q modification has been reported in aspen (GenBank: EEE81588.1) but not in Arabidopsis, suggesting that Q should be found in hybrid aspen but not in *Arabidopsis*. acp^3^U[48,49] and k^2^C[50,51], which have been reported in sequenced chloroplast tRNA were not detected either in total tRNA from 14 d whole seedlings of Arabidopsis. This may be because these modified nucleosides are present in only a few chloroplast tRNA, meaning that their abundance is below the detection threshold for the method used in this study. tRNA enrichment from chloroplast compartments might allow us to see these modifications more easily. Generally patterns of modified nucleosides in plants are similar to those observed in yeast and calf liver, however, several modified nucleosides, including mcm^5^U, mcm^5^s^2^U, Y and Y_OH _that have been detected in yeast individual tRNA species were not detected in plant tRNAs, potentially because of extremely low abundance. Two prokaryotic modified nucleosides, m^2^A and ms^2^io^6^A, were present in plant tRNAs. It is likely that these are from chloroplast or mitochondria subcellular compartments. m^3^C is present in yeast tRNA but was not observed in the tRNAs in this study. At present we are unable to conclude whether this is because m^3^C is present in low abundance or that plants lacks genes responsible for m^3^C modification. Four novel nucleosides were discovered in plant tRNAs. In subsequent work, we will further characterize the identities of these compounds by use of combined LC-MS method.

For the 21 known modified nucleosides mentioned above, we used a loss-of-function study and identified five genes responsible for four specific modified nucleosides, m^1^G (AtTRM10), m^2^G (AtTRM11), m^7^G (AtTRM82) and ncm^5^U (AtKTI12 and AtELP1). Modified nucleosides participate in fine-tuning the activity of tRNAs during translation. For example, defects of certain tRNA modifications result in decreased translation efficiency and increased translational error. Depending on which codon the tRNA recognizes and the codon context, various aspects of cellular metabolism and signaling pathways may be altered. Two of the Arabidopsis tRNA modification mutants identified in this study showed an early flowering phenotype (AtTRM11 mutant) and had reduced organ growth (AtELP1 mutant), respectively. To study the mechanism of early-flowering of AtTRM11 mutant, we will investigate expression of some flowering key regulators (e.g. GI, FT, LFY, FLC and SOC1) to see which pathway is affected in the mutant. Flowering regulation in Arabidopsis is rather complicated network but investigating expression of the key regulators will help to unravel the molecular mechanism. AtELP1 has been reported being involved in different developmental processes (leaf and root elongation) and stress response (anthocyanin biosynthesis and oxidative stress key regulators); we will study gene expression and regulation in the different pathways. With more genes to be identified in the future, we expect to see a more complex profile of the function of modified nucleosides in plant growth, development and stress responses. To understand the molecular mechanisms involved, we need to investigate the temporal and tissue specific expression of tRNA modification enzymes, to find the targets of genes underlying the phenotypic changes of each mutant. In addition to regulation on a translational level, certain Arabidopsis tRNA modifying enzymes might also interact physically with other proteins.

In this study we have established a method for the analysis of modified nucleosides in transfer RNA from plants. With this method we identified five genes responsible for specific modified nucleosides. The advantage of the HPLC method is that we could observe global changes of all the modified nucleosides. The disadvantage is that the sensitivity of the HPLC method we are using could not detect very low abundance modified nucleosides that may be present in only on a few tRNA molecules and the HPLC method alone could not determine the identity of unknown compounds. However, in future experiments we should be able to overcome these problems by enriching for individual tRNA species using hybridization-based Dynabead technology followed by HPLC analysis; and by combining the HPLC with LC-MS or by enriching the compound of interest by HPLC followed by MS or NMR studies to resolve the structure. With the method established in this study, more tRNA modification genes in plants should be able to be identified. Characterization of mutants for these genes will reveal the function of modified nucleosides in plant physiology and gene expression.

## Conclusions

In this study we established a method for analyzing modified nucleosides of tRNA from plant tissues, described the amount and recovery efficiency for each step. We detected 21 modified nucleosides in young seedlings of Arabidopsis and from young tissues of hybrid aspen (*Populus tremula ***× **tremuloides). More importantly, we have predicted and summarized the tRNA modification candidate genes in plants. Through loss-of-function studies we identified five genes responsible for four specific modified nucleosides in *Arabidopsis thaliana*: AtTRM10 for m^1^G, AtTRM11 for m^2^G, AtTRM82 for m^7^G, AtKTI12 and AtELP1 for ncm^5^U modification. We conclude with the method established here, more modified nucleosides in plants can be investigated in order to understand the function of tRNA modifications in plant growth, development and stress responses. This systematic study on tRNA modification genes in Arabidopsis is very useful as a tool for those in the same research area and those who are interested in developing new research projects related to nucleoside modification on small RNAs. Also the four novel plant-specific modified nucleosides will be of great interest for plant researchers.

## Methods

### Plant growth conditions

For total RNA preparation from young seedlings *Arabidopsis thaliana *ecotype Col.0 was grown as a lawn in soil and vermiculite (3:1) in a greenhouse at 22°C/18°C (day/night temperature), with light intensity of 150 μmol m^-2 ^s^-1 ^and 60% humidity under long-day conditions (16 h-light/8 h-dark cycle). 3-weeks seedlings were harvested and frozen in liquid nitrogen for subsequent RNA extraction. Hybrid aspen (*Populus tremula ***× ***tremuloides*; clone T89) were grown in soil in green house at 22/18°C (day/night temperature), with light intensity of maximum 400 μmol m^-2 ^s^-1 ^from natural daylight (controlled by curtains, supplemented when required with high-pressure sodium lamps) and 80% humidity under long-day conditions (16 h-light/8 h-dark cycle). Young leaves and apical shoot tips within 2 cm from the top of about 1.5 m high trees were collected and frozen in liquid nitrogen for RNA extraction.

For phenotypic study, seeds for all lines were stratified 3 days in +4°C before being sown; plants were grown in LD conditions (16 hr photoperiod, light density 150 μmol m^-2 ^s^-1^, day temperature 22°C, night temperature 18°C, and 60% humidity). MS (Murashige-Skoog, Duchefa Biochemie) medium, supplemented with 0.8% plant agar (Duchefa Biochemie) and 1% sucrose (Sigma) was used for Arabidopsis root growth studies under LD conditions (same as above).

All T-DNA mutant lines were purchased from The European Arabidopsis Stock Center (NASC, http://arabidopsis.info/). Homozygote or heterozygote genotypes were determined using gene specific primers designed by T-DNA primer design tool (Salk Institute Genomic Analysis Laboratory, http://signal.salk.edu/tdnaprimers.2.html), LBa1 primer (5'-TGGTTCACGTAGTGGGCCATCG-3') was used as left border primer for T-DNA insertion.

### RNA isolation and digestion

Total RNA was extracted using Trizol Reagent (Invitrogen), and RNA concentration was determined using NanoDrop ND-1000 spectrophotometer (Thermo Scientific). sRNAs (including tRNA, miRNA and snRNA) were separated from rRNA and mRNA using the LiCl method: rRNA and mRNA were precipitated with 2 M LiCl final concentration, sRNAs in supernatant were precipitated with 3 volumes of ethanol, washed once with 70% ethanol and dissolved in 0.1 M Tris pH7.4, 0.1 M NaCl. tRNA was further purified using DE52 anion exchange resin: RNA in binding buffer (0.1 M Tris pH7.4, 0.1 M NaCl) was loaded on DE52 column (bed volume 2 ml), washed three times with 5 ml binding buffer each time, eluted with 7 ml elution buffer (0.1 M Tris pH7.4, 1 M NaCl). tRNA was precipitated with isopropanol, washed with 70% ethanol and dissolved in MQ water. 50 μg tRNA from Arabidopsis or hybrid aspen were degraded to nucleosides with P1 nuclease (Yamasa Corporation, Japan) and bacterial alkaline phosphatase (Sigma) as following: to 50 μg tRNA (in 100 μl MQ) add 10 ul of 20 mM ZnSO_4_, 10 ul nuclease P1 (1 mg/ml, 200 units/mg in 30 mM NaAc pH5.3) and digest at 37°C for at least 24 hrs; add 5 ul bacterial alkaline phosphatase (about 190 units/ml, 30 units/mg, diluted 1:100 with water) and 20 ul 0.5 M Tris pH8.3 and digest at 37°C for 2 hrs, the digested nucleosides are now ready for HPLC analysis.

### HPLC analysis

Modified nucleosides were analyzed using Reverse-phase HPLC (Waters Alliance HPLC system and Waters Absorbance Detector 2996; Waters, http://www.waters.com) and C-30 column (Develosil C-30 reverse-phase column, 250 × 4.6 mm; Phenomex Ltd.). The buffer gradient was as follows: buffer A (0.01 M NH_4_H_2_PO_4_+2.5% MeOH, pH5.3), buffer B (0.01 M NH_4_H_2_PO_4_+20% MeOH, pH5.1), buffer C (0.01 M NH_4_H_2_PO_4_+35% Acetonitril). 0-12 min, 100% buffer A; 12-20 min, 100% A; 20-25 min 90% buffer A, 10% buffer B; 25-32 min, 75% buffer A, 25% buffer B; 32-36 min 40% buffer A, 60% buffer B; 36-45 min 38% buffer A, 62% buffer B; 45-80 min 100% buffer B; 80-87 min 100% buffer C; 87-95 min, 100% buffer A. The threshold level for detection of modified nucleosides was approximately 0.002% of the total area. The abundance of each modification was calculated relative to two internal standards (Ψ and t^6^A), with similar results.

### Bioinformatics analysis of plant tRNA modifying genes

The protein sequences for tRNA modifying genes from *S. cerevisiae *or *E. coli *were used to find homologous genes in *Arabidopsis thaliana *using blastp tool (on TAIR database: The Arabidopsis Information Resource, http://www.arabidopsis.org), cut-off value 1e-06 (except for KTI13 and SUA5 where cut-off value is 1e-05). Other plant homologs were identified using the Arabidopsis genes as query sequence, using the tblastn program and the NCBI nucleotide collection (nr/nt) database with default settings, the cut-off value was above 60% positives and e-value above 1e-60. All plant genes were aligned with multiple sequence alignment using the CLUSTAW program http://align.genome.jp/. The unrooted neighbour-joining tree was constructed using Geneious Basic 4.5.5 Tree Builder http://www.geneious.com. Data for Arabidopsis gene expression in different tissues and under different developmental stages was downloaded from AtGenExpress database http://jsp.weigelworld.org/expviz/expviz.jsp. The developmental and tiling datasets were downloaded from the Tileviz database http://jsp.weigelworld.org/tileviz/tileviz.jsp. Mean-normalized expression values were transformed into log values. The heat maps were constructed using the MeV (MultiExperiment Viewer) v.4.3.02 software using default parameters.

Subcellular localization of proteins encoded by Arabidopsis tRNA modification candidate genes were predicted using three different programs: TargetP http://www.cbs.dtu.dk/services/TargetP/; WoLFPSORT http://wolfpsort.org/ and ESLpred http://www.imtech.res.in/raghava/eslpred/ with default settings for plant organisms, hybrid approach were chosen for ESLpred.

## Abbreviations

m^1^A: 1-methyladenosine; m^2^A: 2-methyladenosine; m^6^A: N6-methyladenosine; ms^2^io^6^A: 2-methylthio-N6-(cis-hydroxyisopentenyl)adenosine; t^6^A: N6-threonylcarbamoyladenosine; m^6^t^6^A: N6-methyl-N6-threonylcarbamoyladenosine; i^6^A: N6-isopentenyladenosine; ms^2^t^6^A: 2-methylthio-N6-threonyl carbamoyladenosine; Ar (p): 2'-O-ribosyladenosine (phosphate); m^3^C: 3-methylcytidine; m^5^C: 5-methylcytidine; ac^4^C: N4-acetylcytidine; m^1^G: 1-methylguanosine; m^2^G: N2-methylguanosine; m^7^G: 7-methylguanosine; m^2^_2_G: N2, N2-dimethylguanosine; Y: wybutosine; Y_OH_: hydroxywybutosine; o^2^Y: peroxywybutosine; I: inosine; m^1^I: 1-methylinosine; Ψ: pseudouridine; Ψm: 2'-O-methylpseudouridine; D: dihydrouridine; mcm^5^U: 5-methoxycarbonylmethyluridine; mcm^5^s^2^U: 5-methoxycarbonylmethyl-2-thiouridine; s^2^U: 2-thiouridine; acp^3^U: 3-(3-amino-3-carboxypropyl) uridine; m^5^U: 5-methyluridine; ncm^5^U: 5-carbamoylmethyluridine; Q: queuosine; manQ: mannosyl-queuosine; τm^5^U: 5-taurinomethyluridine; τm^5^s^2^U: 5-taurinomethyl-2-thiouridine; Um: 2'-O-methyluridine; Cm: 2'-O-methylcytidine; Am: 2'-O-methyladenosine; Gm: 2'-O-methylguanosine

## Authors' contributions

PC carried out the T-DNA mutant screen and phenotype identification, participated in method set up for modified nucleoside analysis of young seedlings of Arabidopsis and hybrid aspen and drafted the manuscript. GJ carried out HPLC analysis of modified nucleosides of all mutants and wild type Arabidopsis and hybrid aspen. BZ conceived of the study, participated in bioinformatic analysis and helped to draft the manuscript. All authors read and approved the final manuscript.

## Note

**RNA modification database **http://library.med.utah.edu/RNAmods/

**NCBI database **http://www.ncbi.nlm.nih.gov/

**European Arabidopsis Stock Center **[NASC, http://arabidopsis.info/]

**Arabidopsis microarray database TileViz **http://jsp.weigelworld.org/tileviz/tileviz.jsp

**Pfam database **http://pfam.sanger.ac.uk/

**AtGenExpress database **http://jsp.weigelworld.org/expviz/expviz.jsp

**The Arabidopsis Information Resource **http://www.arabidopsis.org

**CLUSTAW program **http://align.genome.jp/

**Waters company **http://www.waters.com

**TargetP program **http://www.cbs.dtu.dk/services/TargetP/

**WoLFPSORT program **http://wolfpsort.org/

**ESLpred program **http://www.imtech.res.in/raghava/eslpred/

**SIGnAL T-DNA Primer Design Tool **http://signal.salk.edu/tdnaprimers.2.html

## Supplementary Material

Additional file 1 **Phylogenetic trees of plant tRNA modification candidate genes**. All protein sequences were aligned using CLUSTAW multi-sequence alignment program http://align.genome.jp/, non-rooted neighbourhood-joining tree was constructed using Geneious 4.5.5 software (see Methods) for each group of plant genes. For query gene from *S. cerevisiae *or *E. coli*, tree is named according to the query gene (e.g. TRM11 tree). Candidate genes were annotated with accession number and name of the organism. Click here for file

Additional file 2 **Subcellular prediction of tRNA modification candidate proteins**. Protein sequence of Arabidopsis tRNA modification candidate genes in Table 3 were used in three different protein subcellular localization prediction programs: TargetP, WoLFPSORT and ESLpred. Predicted subcellular localization were also shown in Table 3 when at least two program gave similar predictions. Click here for file
