# Supplementary material for: Transfer RNA modifications and genes for modifying enzymes in Arabidopsis thaliana
Source: BMC Plant Biol. 2010 Sep 14;10:201. doi: 10.1186/1471-2229-10-201 (PMC2956550; doi:10.1186/1471-2229-10-201)

**Supplemental Data**

All protein sequences were aligned using CLUSTAW multi-sequence alignment program (http://align.genome.jp/sit-bin/clustalw), unrooted Neighbour-Joining tree was constructed using Geneious 4.5.5 software.

Dus tree


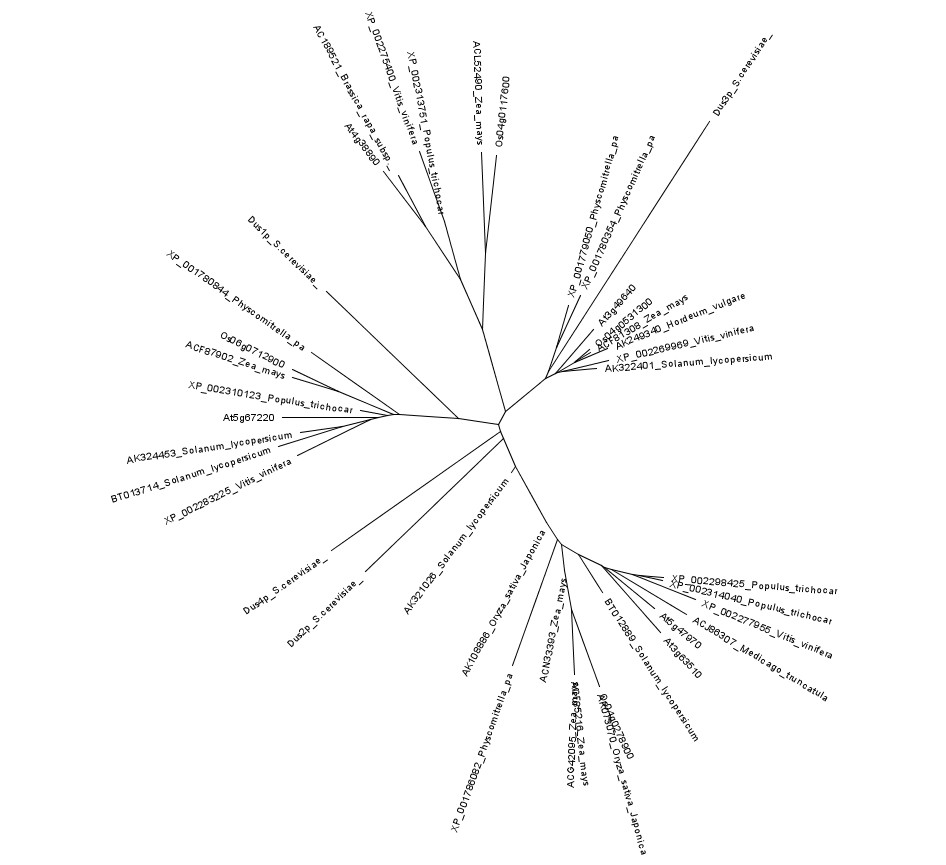


Pus tree


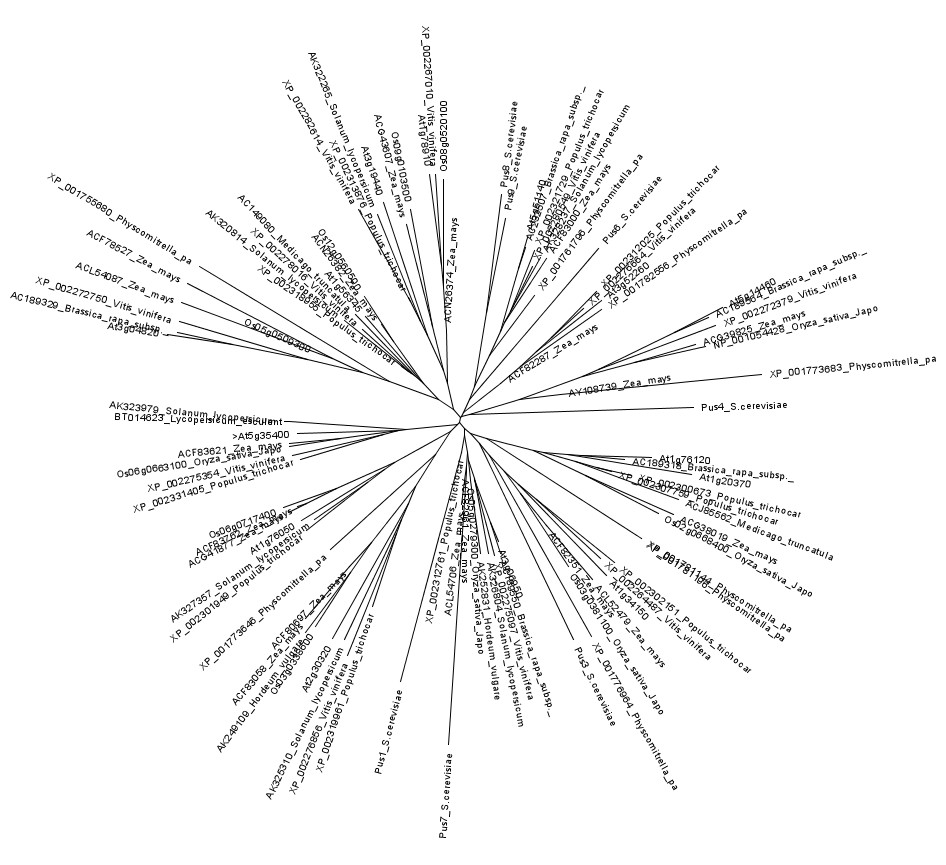


Sit4 gene homologs in Arabidopsis


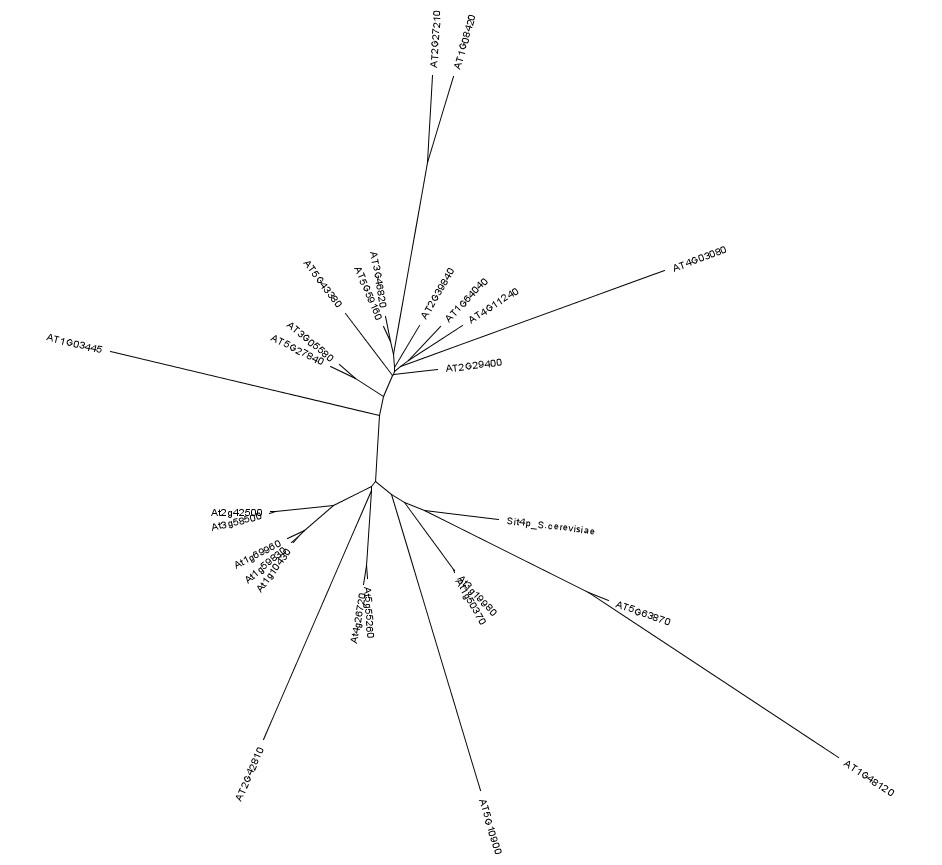


Sap tree


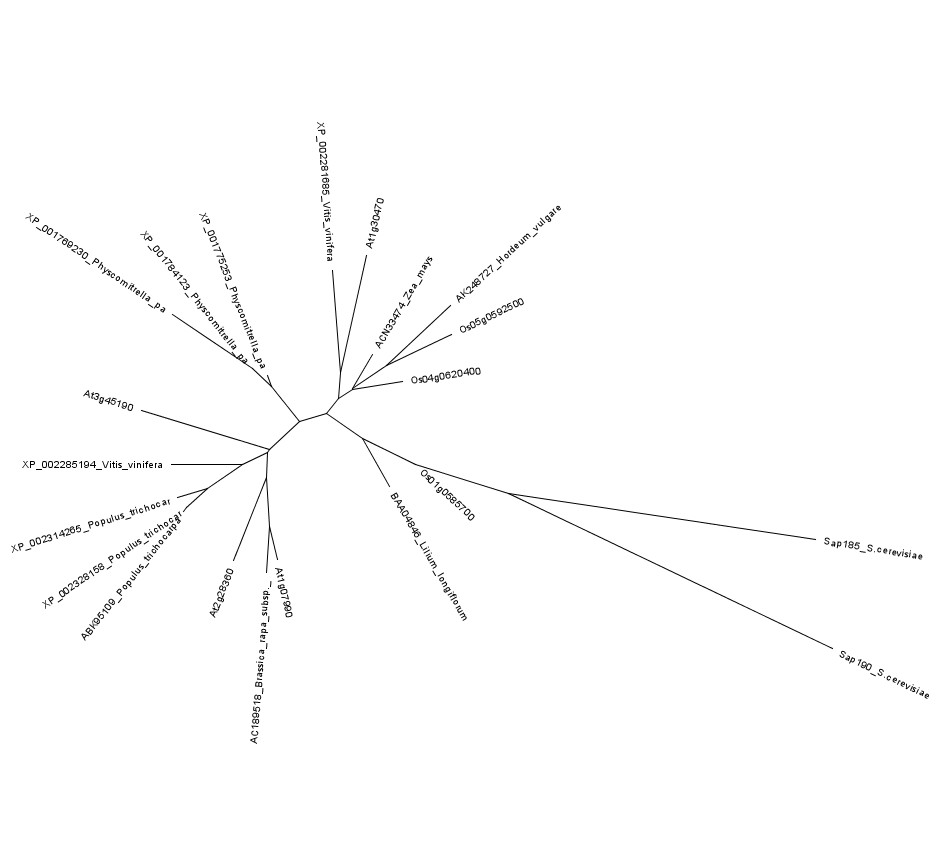


Kti11 tree

**
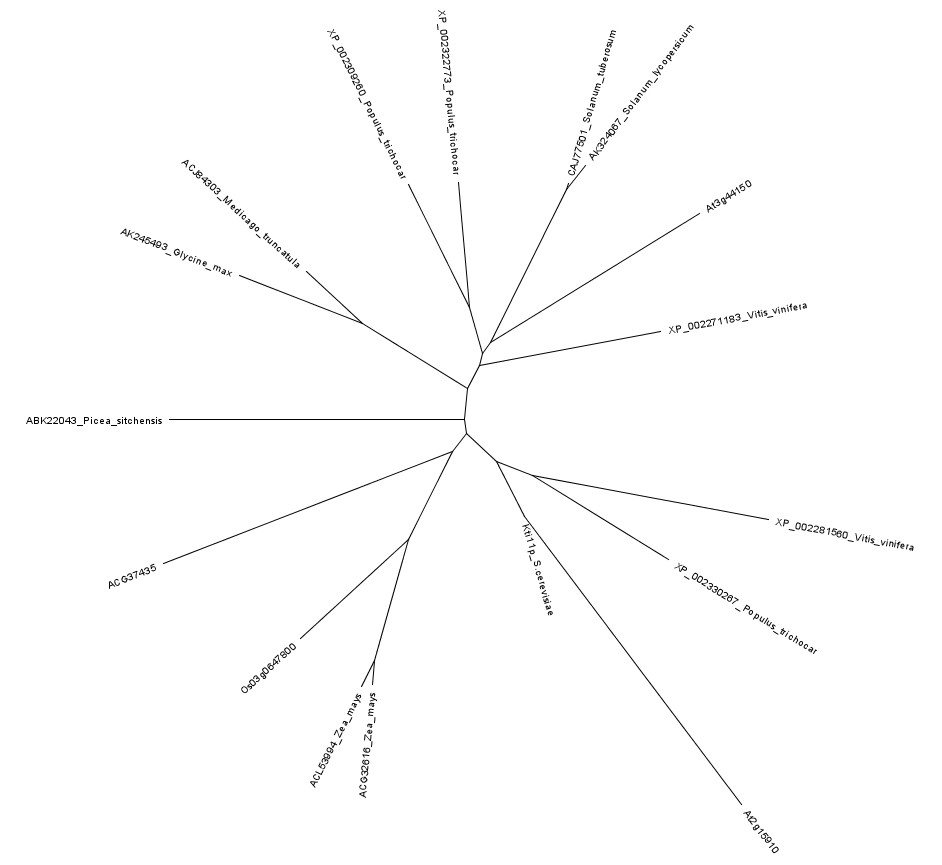
**

Kti13 tree

**
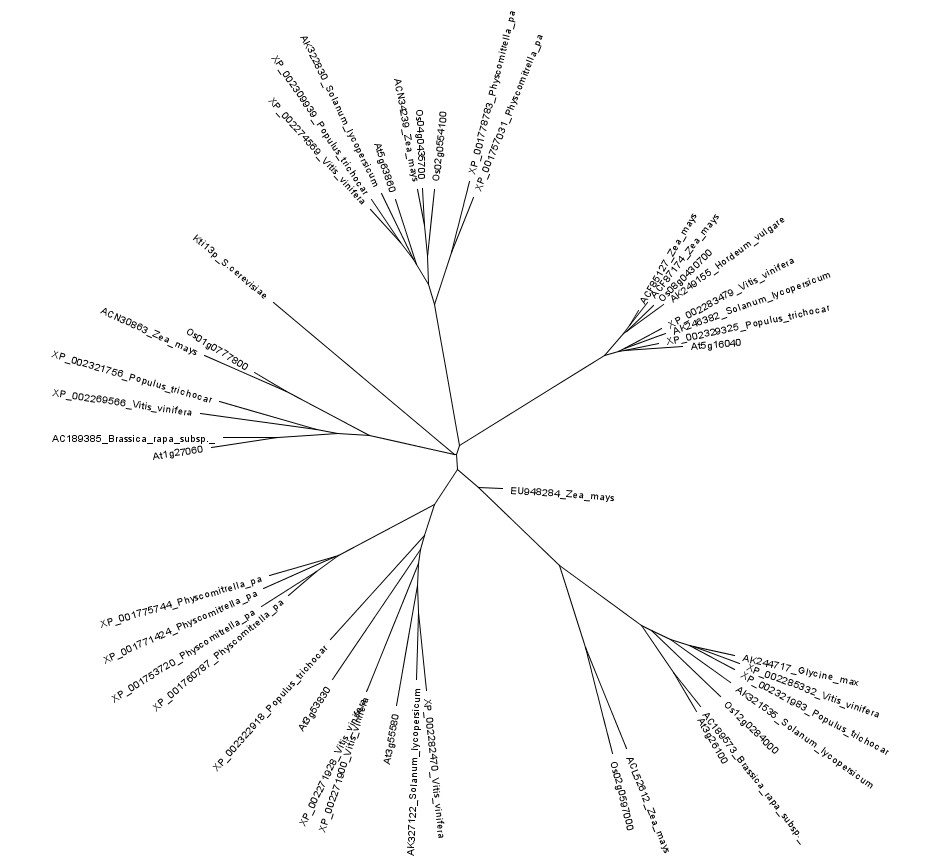
**

Trm61 tree


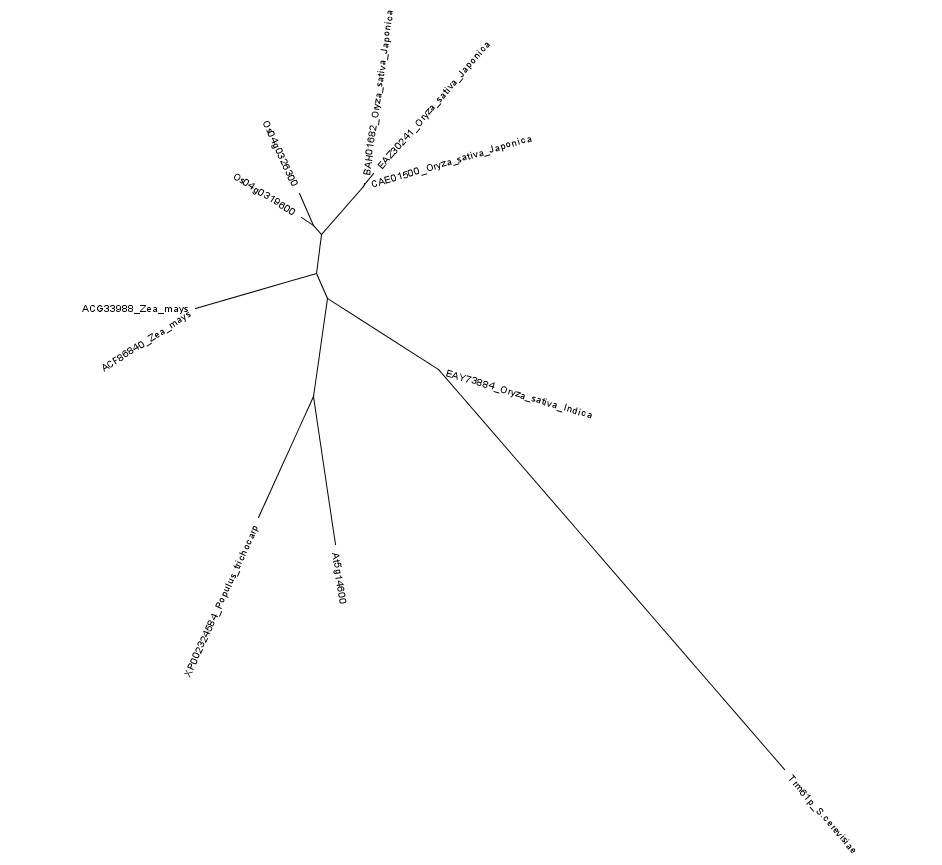


Trm6 tree


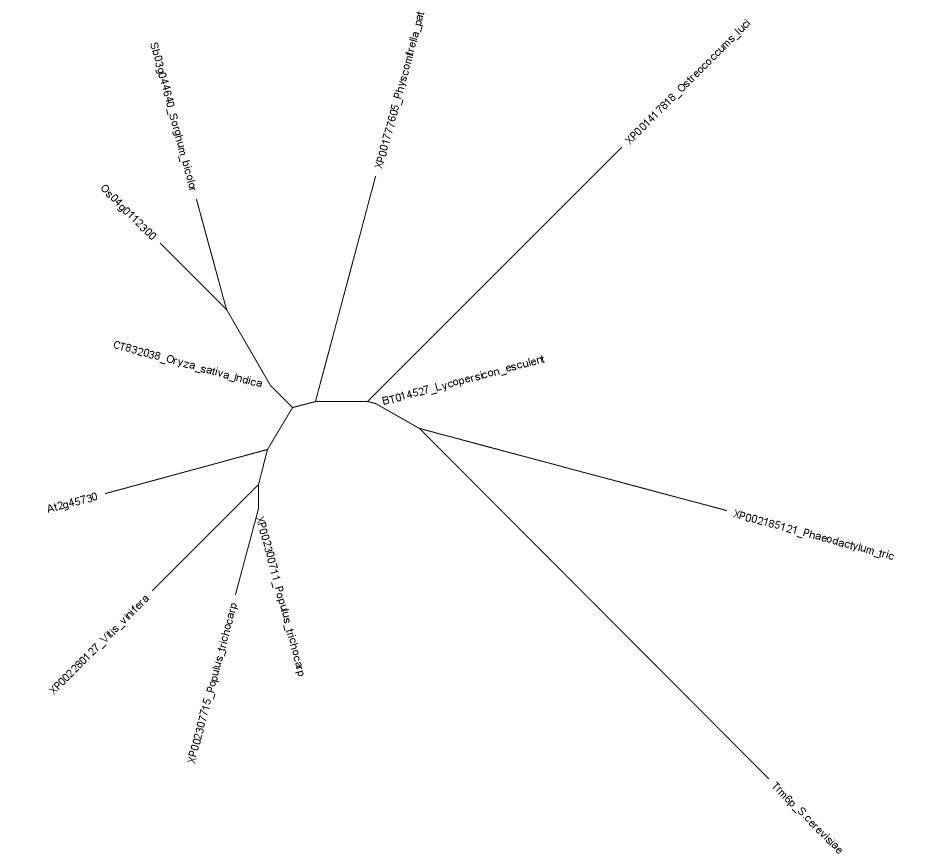


Trm4 tree

**
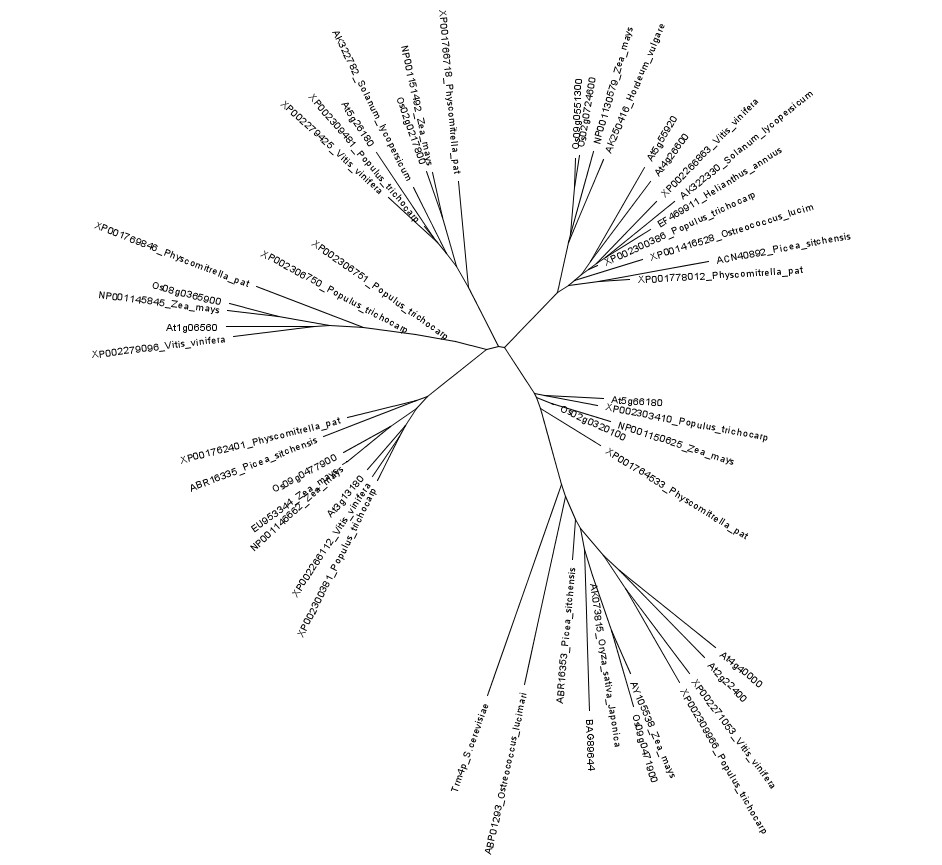
**

Trm7 tree


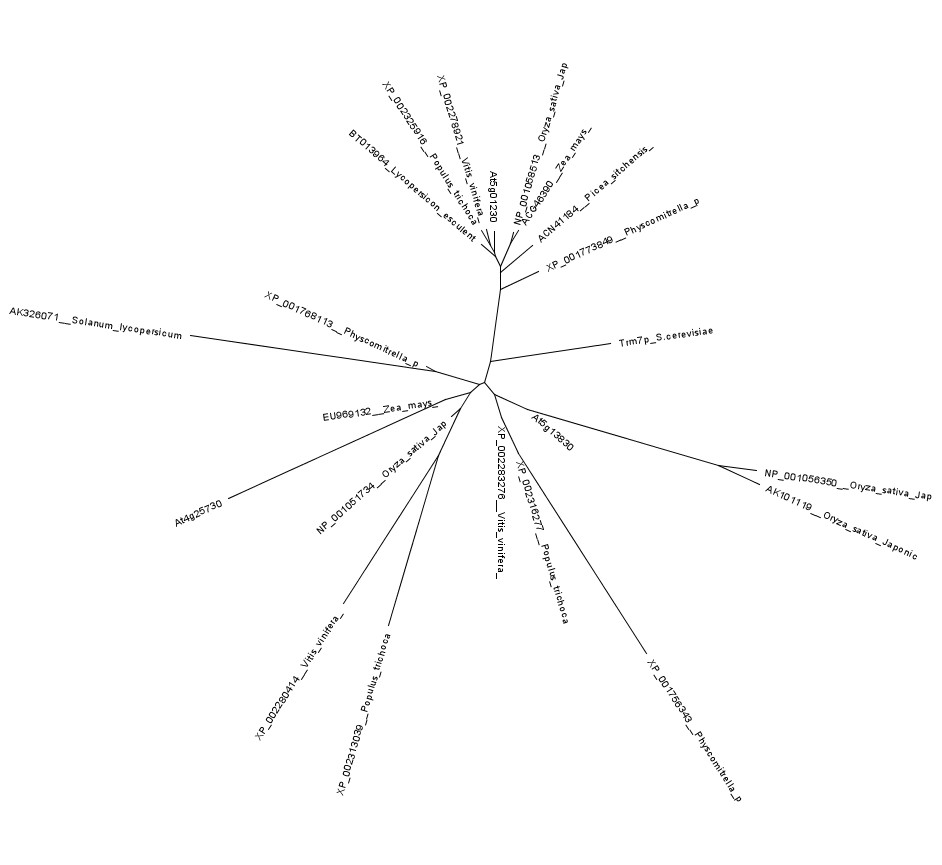


Trm8 tree


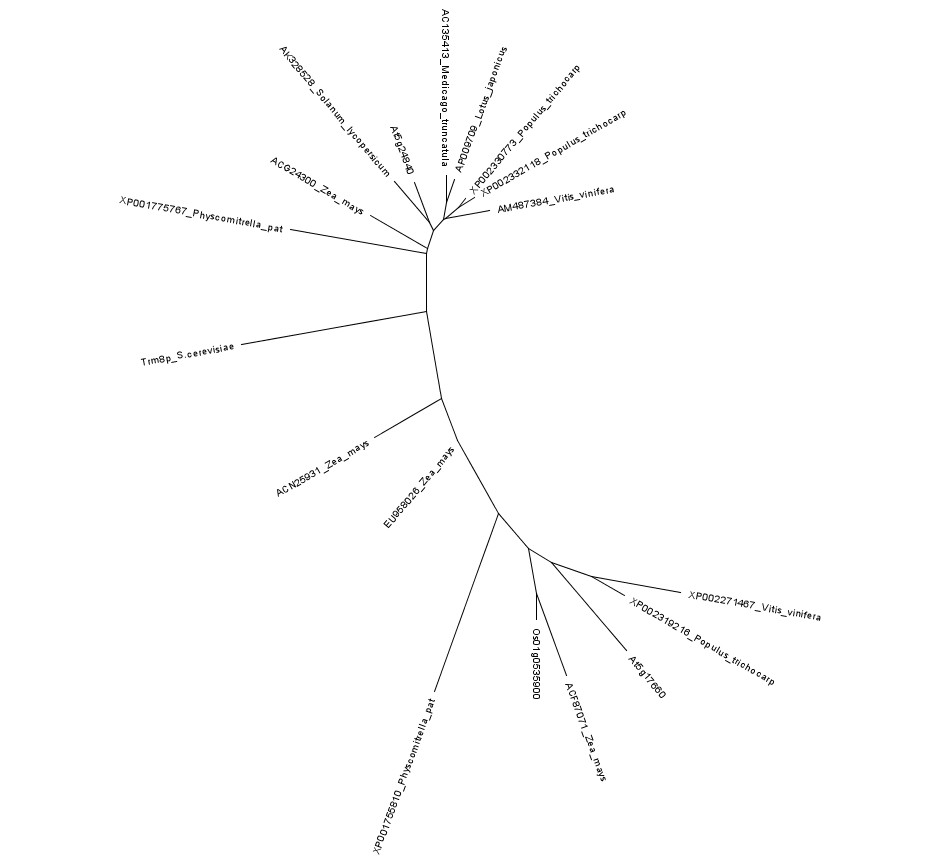


Trm2 tree


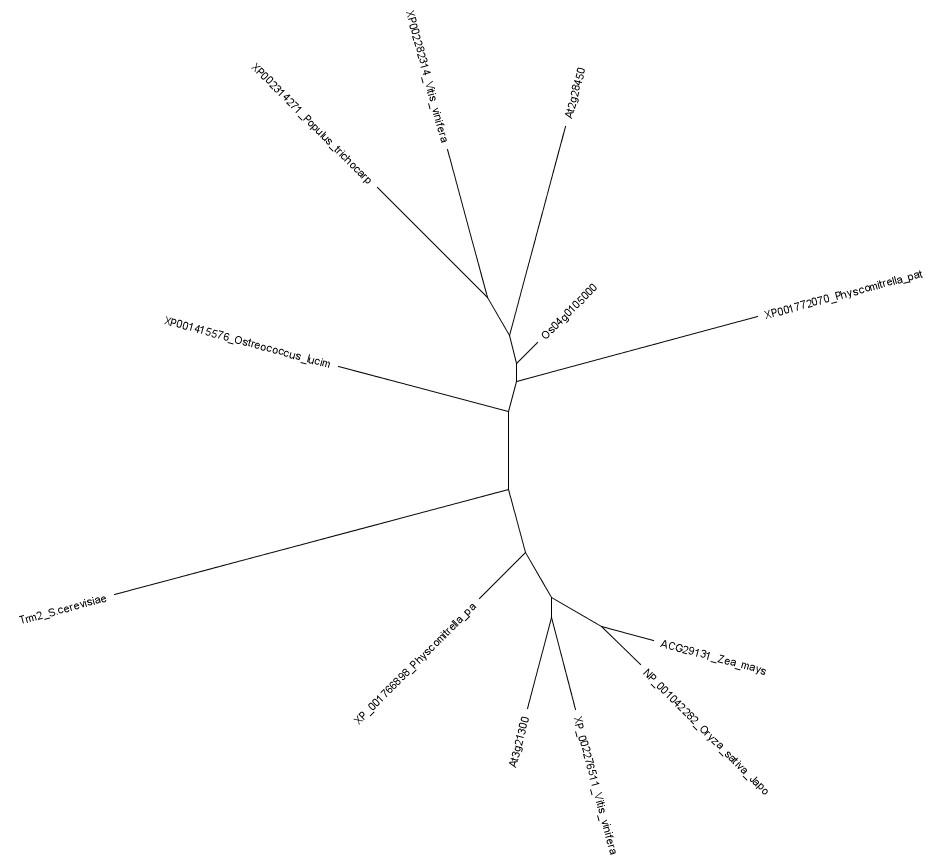


Tad tree


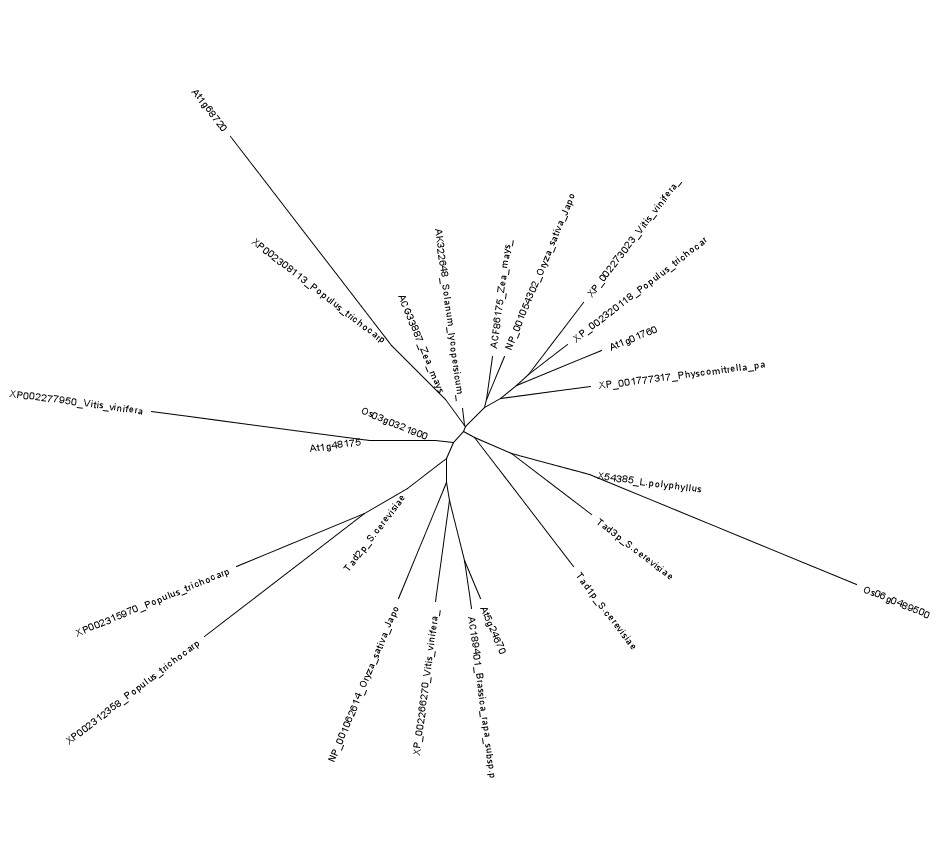


Trm5 tree


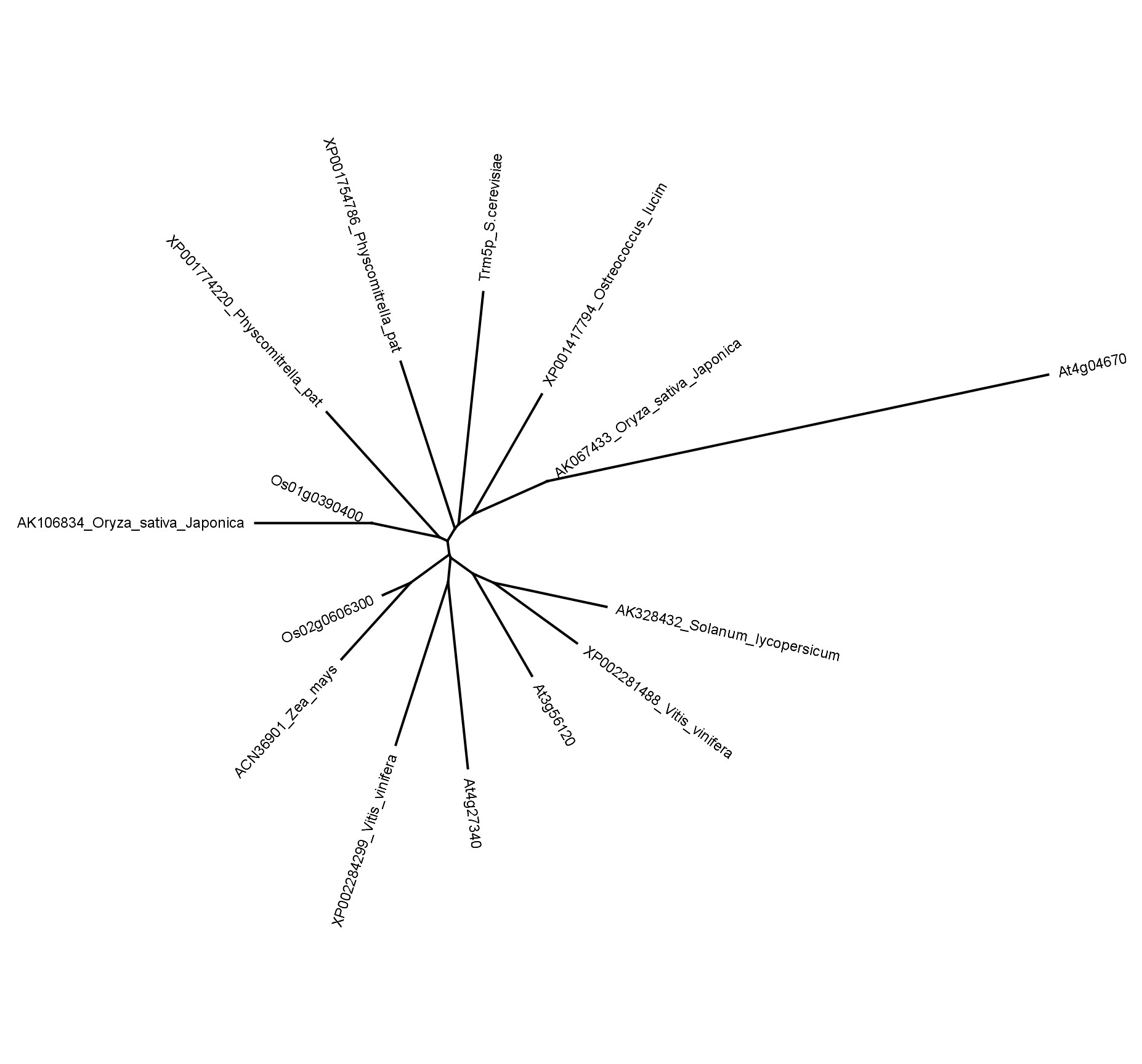


Trm3 tree


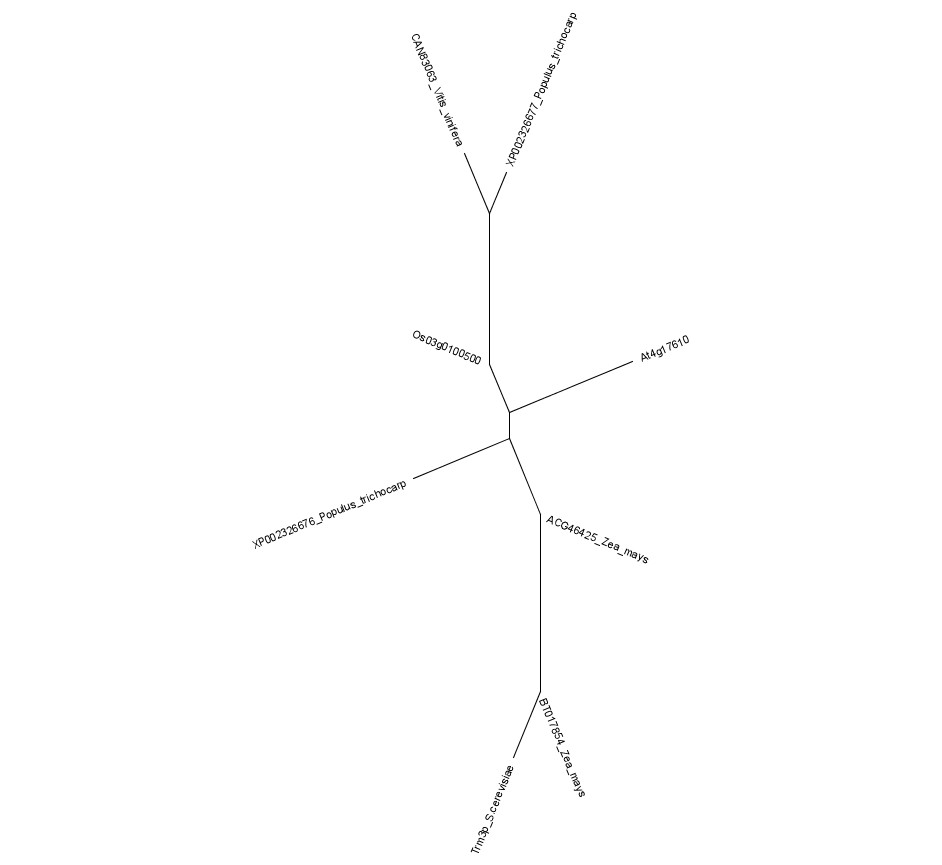


Trm112 tree


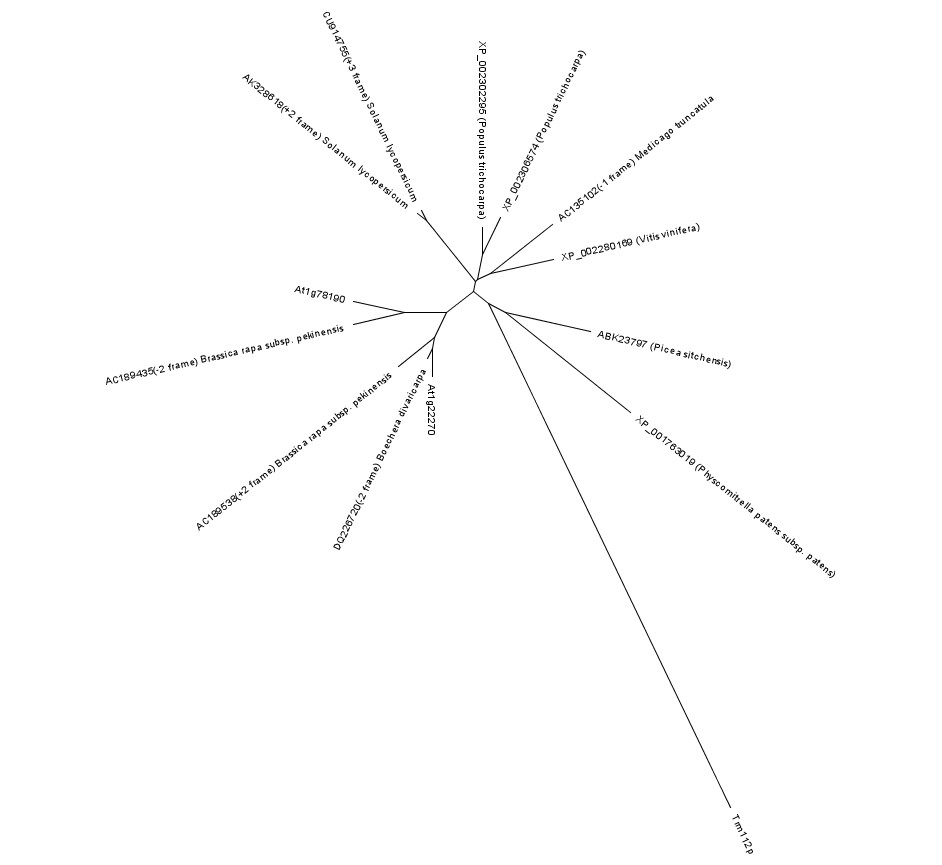


Tan1 tree


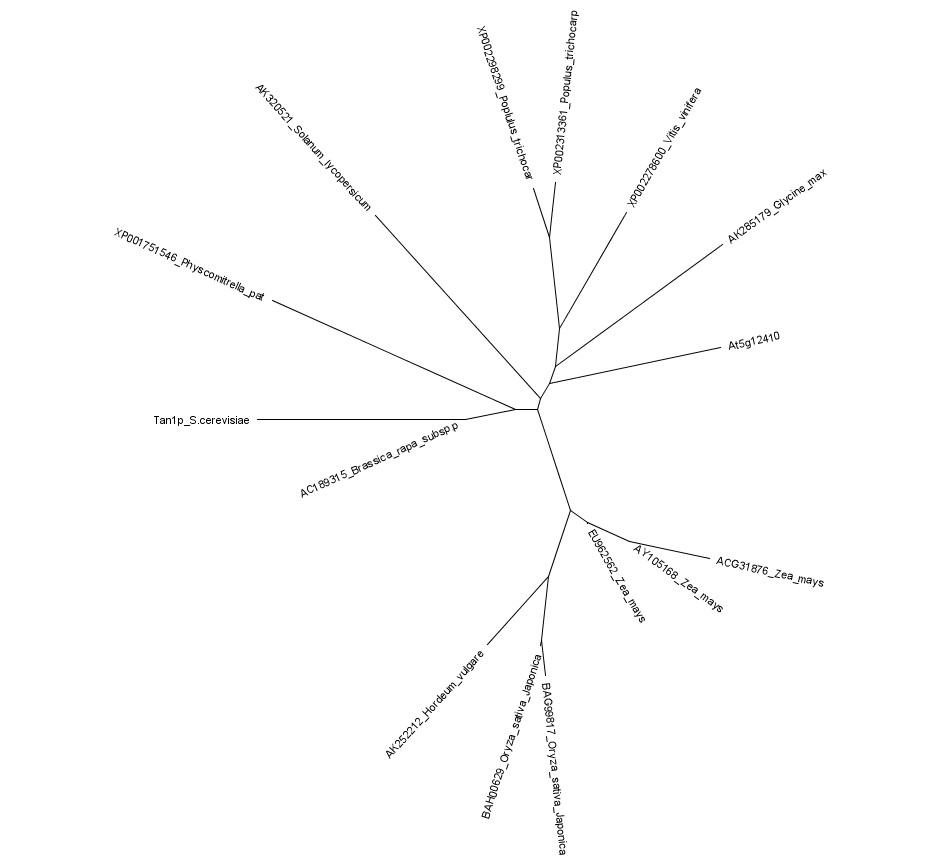


Trm1 tree


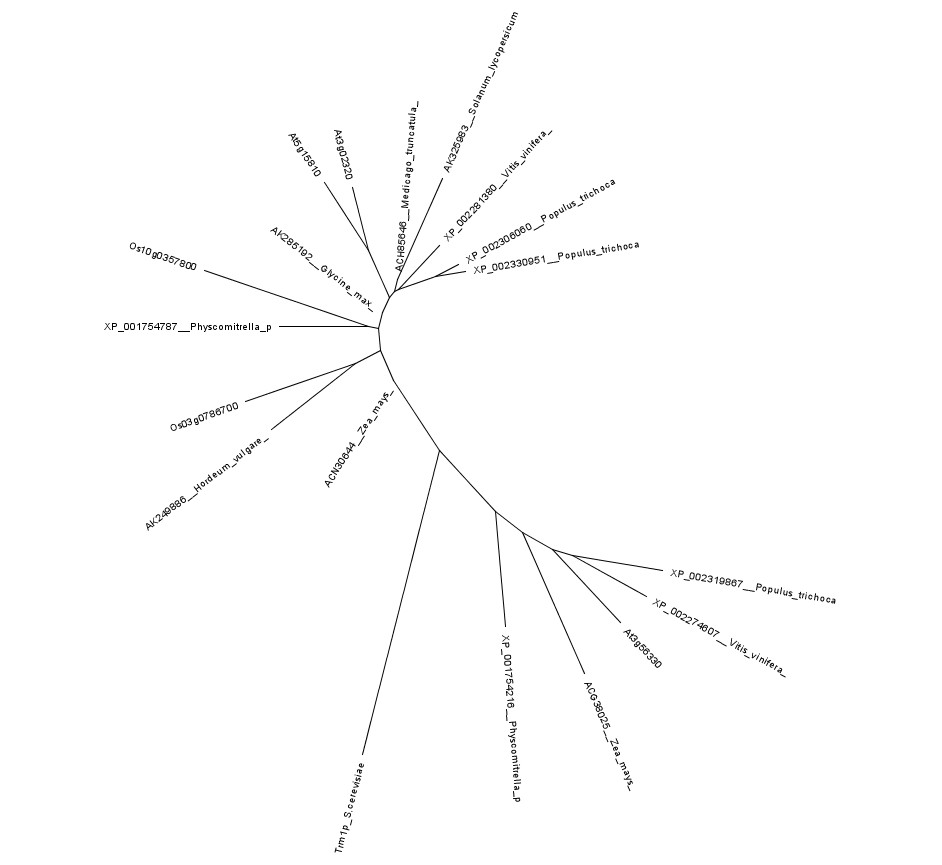


Trm13 tree


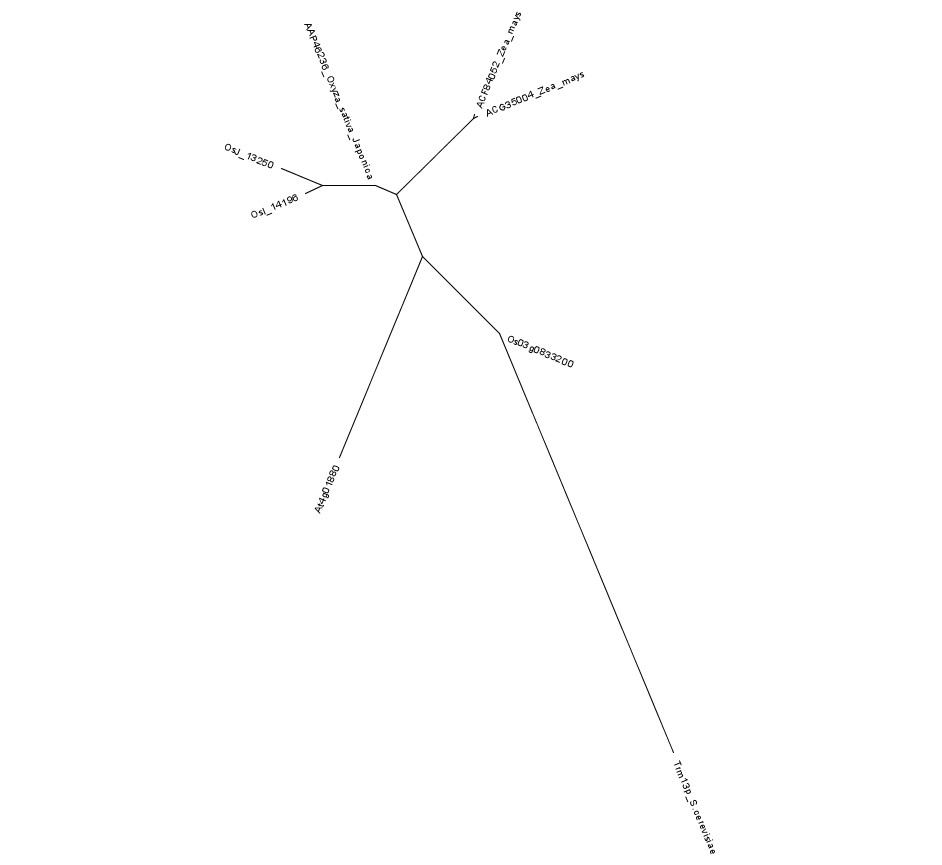


Sua5 tree


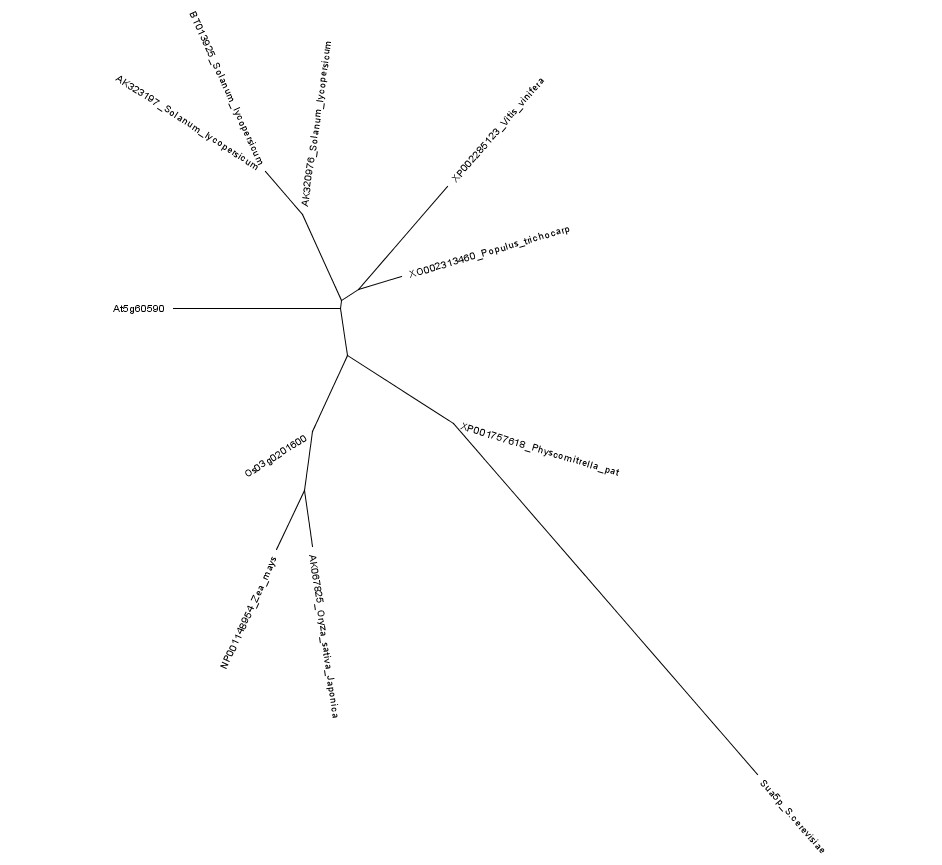


MiaB tree


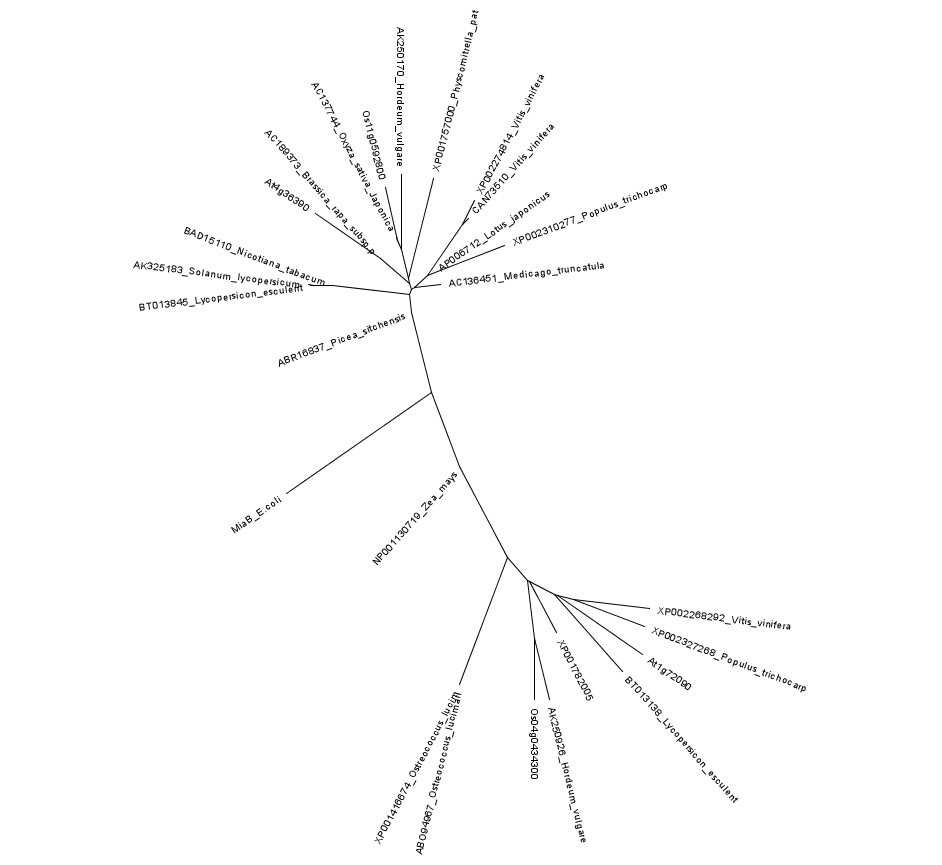


MOD5 tree


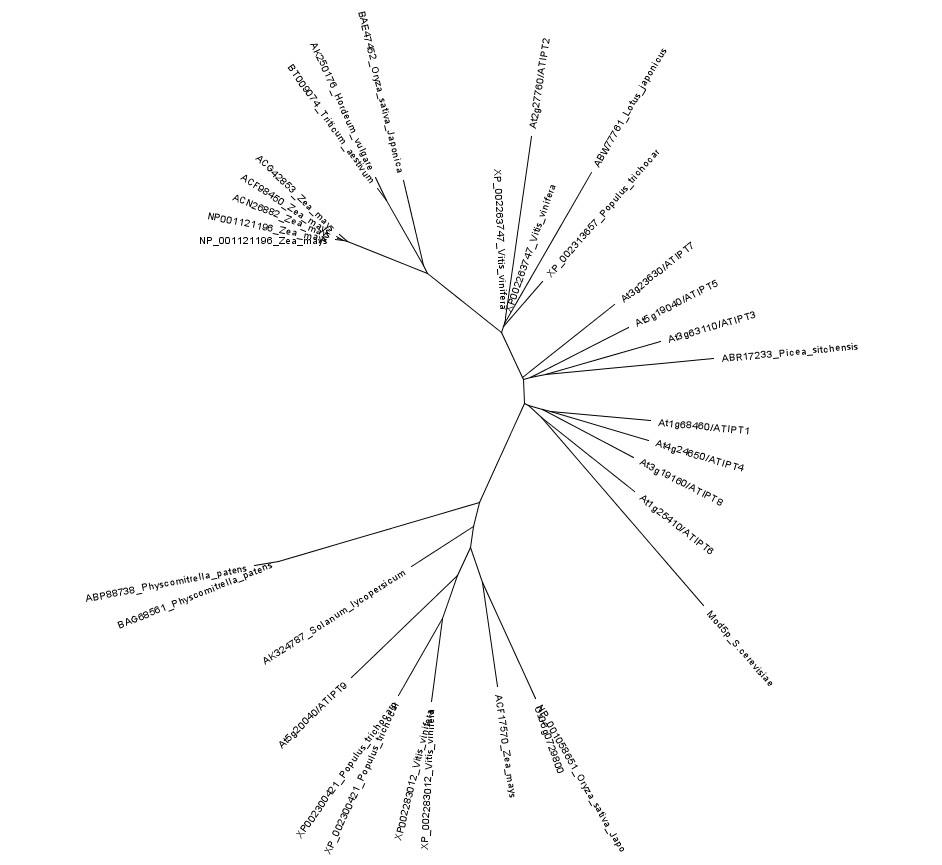

Supplement: Additional file 1 — Phylogenetic trees of plant tRNA modification candidate genes. All protein sequences were aligned using CLUSTAW multi-sequence alignment program http://align.genome.jp/, non-rooted neighbourhood-joining tree was constructed using Geneious 4.5.5 software (see Methods) for each group of plant genes. For query gene from S. cerevisiae or E. coli, tree is named according to the query gene (e.g. TRM11 tree). Candidate genes were annotated with accession number and name of the organism. [file 1471-2229-10-201-S1.DOC]
